# Quantitative Characterization of RNA-Splinted Ligation as a Multipurpose Post-Synthetic Strategy for High-Density DNA Microarrays Synthesized by Nucleic Acid Photolithography

**DOI:** 10.1021/acs.analchem.6c03811

**Published:** 2026-09-17

**Authors:** Arya Das, Mark M. Somoza

**Affiliations:** † 9184 Technical University of Munich, Garching 85748, Germany; ‡ TUM School of Natural Sciences, Garching 85748, Germany; § 27258 Leibniz Institute for Food Systems Biology at the Technical University of Munich, Freising 85354, Germany; ∥ Chair of Food Chemistry and Molecular Sensory Science, Technical University of Munich, Freising 85354, Germany; ⊥ Institute of Inorganic Chemistry, Faculty of Chemistry, University of Vienna, Vienna 1090, Austria

## Abstract

Complex arrays of surface-immobilized DNA oligonucleotides are widely used across diverse fields of bioanalytics, particularly for their applications in massively parallel genomic assays ranging from spatial transcriptomics to protein–DNA recognition studies. DNA microarrays produced by photolithography enable the introduction of versatile chemistries and complexities to surfaces with complete spatial control, but are partially constrained by synthetic error rates for longer probe architectures and by incompatibility with photosensitive chemical building blocks commonly used in phosphoramidite chemistry. Here, we present RNA-splinted ligation as a multipurpose post-synthetic microarray fabrication strategy for extending the complexity of DNA on microarray surfaces. We systematically characterize the parameters governing on-array DNA ligation and demonstrate its applications in labeling, reverse transcription, and the generation of highly complex patterns by introducing an otherwise incompatible photochemistry.

## Graphical abstract

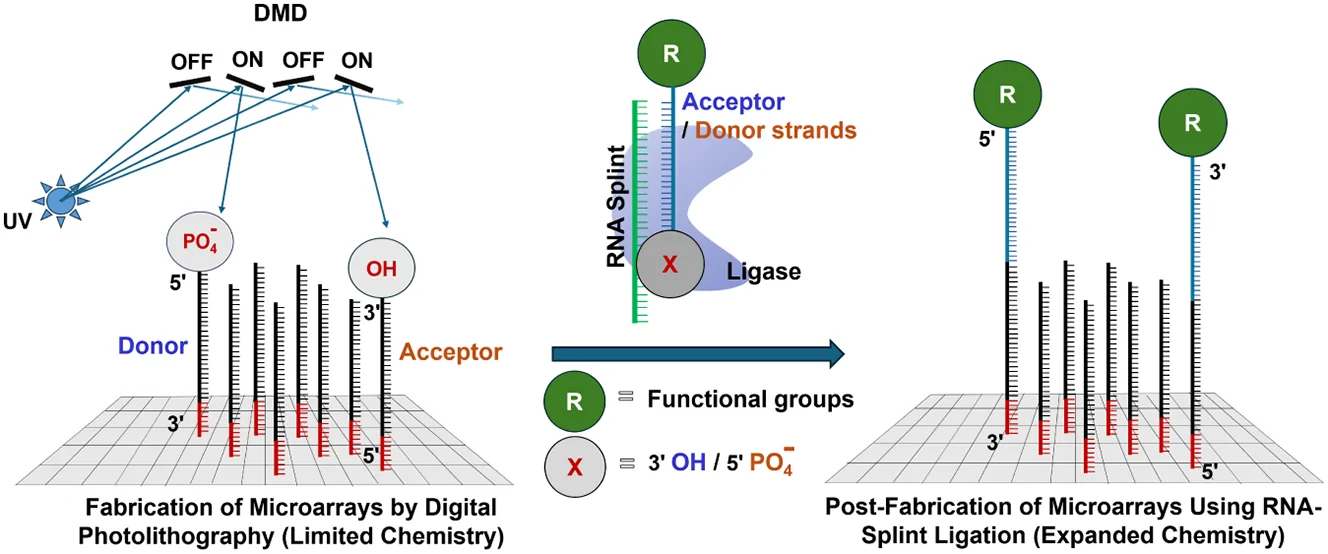

## Introduction

Maskless photolithography is a low-cost approach for large-scale synthesis of nucleic acids with very high chemical versatility. DNA and RNA microarrays,^1–5^ as well as microarrays containing noncanonical or xenobiotic nucleic acids,^6–9^ have found applications in multiple areas of research, including nanotechnology, synthetic biology, nucleic acid therapeutics, and genomics. A major strength of microarrays is their ability to support high-throughput assays for the systematic profiling of nucleic acid interactions. Moreover, hybridization and the self-assembling nature of DNA expand their scope for studying cell-free gene expression systems, genetic circuits, and a wide range of biosensing operations. Considerable progress has also been made toward using large-scale oligonucleotide pools for data storage applications, where scalable, low-cost DNA libraries can efficiently encode digital information. The functionality of these platforms can be extended by approaches that go beyond what is achievable with available synthesis building blocks alone. Ligation reactions, which form covalent bonds between nucleic acid strands, can add significant flexibility for introducing otherwise incompatible chemistries and for extending microarray probe length beyond what is feasible or practical using *in situ* synthesis.

We have previously published multiple post-fabrication strategies for microarrays to enhance the functionality of surface oligonucleotides. The photolithographic fabrication itself allows complex layouts with near-arbitrary combinations of phosphoramidite chemistries, such as DNA (3′ → 5′ and 5′ → 3′), and others, including 2′F-ANA,^6^ L-DNA,^10^ RNA,^4^ 2′OMe-RNA,^9^ branching and spacer modifications,^8,9^ phosphorothioate bonds,^11^ and fluorescent phosphoramidites.^12–14^ Post-synthetic modifications have primarily been enzymatic, including DNA double-stranding using polymerases,^15^ 3′ terminus extension using TdT polymerase,^9^ and conversion of DNA arrays to RNA arrays using T7 RNA polymerase, followed by enzymatic degradation of the DNA template.^16^ Ligation adds considerable versatility to the post-synthetic modification toolset by enabling the addition of entire oligonucleotides. Oligonucleotide addition can also be performed using click chemistry,^17,18^ which potentially has a higher yield but introduces unnatural linkages that may alter the affinity or function of DNA-binding proteins or enzymes. The lower efficiency of ligation and its sensitivity to mismatches between the splint and the acceptor and donor strands near the junction also favor its use for array-based mismatch detection assays, and for the parallel addition of multiple different oligonucleotides to the array via multiple donor and acceptor oligonucleotides and their corresponding splints.^19,20^

Ligation reactions mediated by either DNA or RNA ligase enzymes join the 5′ phosphate and the 3′ hydroxyl ends of the nucleic acid substrates in the presence of nucleotide cofactors and divalent metal cations.^21^ In contrast, many chemical conjugation strategies require derivatization of the ends, which may be incompatible with the surface derivatization, synthesis chemistry, or the nucleotides themselves.^17,22–25^ Therefore, in the context of DNA microarrays, enzymatic ligation is a potential alternative approach for post-synthetic modification. DNA ligases catalyze the reaction using a three-step adenosine monophosphate (AMP) transfer mechanism, in the presence of nucleotide cofactors such as adenosine triphosphate (ATP) or nicotinamide adenine dinucleotide (NAD^+^). The reaction starts with enzyme adenylation, in which a lysine residue in the ligase’s active site initiates a nucleophilic attack on the α-phosphate of ATP or NAD^+^ to form a phosphoramidate bond and release pyrophosphate or nicotinamide mononucleotide (NMN). This is followed by DNA adenylation, in which the 5′ phosphate oxygen of the nicked DNA substrate attacks the electrophilic phosphorus in the enzyme-bound AMP. This leads to the formation of a pyrophosphate linkage, resulting in a DNA adenylate intermediate. In the last step, phosphodiester bond formation occurs when the 3′-hydroxyl group on the adjacent DNA reacts with the DNA adenylate intermediate, displacing AMP.^26–30^

Splint-mediated ligation reactions provide an efficient route for end-joining single-stranded oligonucleotides (ssDNA) temporarily aligned with DNA or RNA splints.^31–36^ We show that RNA-splinted ligation reactions can be used to join surface-bound ssDNA to an independently synthesized DNA oligonucleotide. The splint enforces the identity of the DNA strands to be joined, and increases ligation efficiency. Following ligation, the RNA splints can be easily removed from the arrays using a simple RNase treatment or by a denaturing formaldehyde wash, leaving the ligated probes immobilized on the surface.

In comparison with DNA-splinted ligation, RNA-splinted ligation offers two advantages in DNA microarray applications. The greater stability of the RNA-DNA heteroduplex leads to shorter on-array splint complementarity, thereby reducing the number of synthesis cycles; and RNase H treatment provides an efficient and gentle method for selective removal of RNA splints after ligation, avoiding harsher denaturing strategies that could damage the array.

Compared to hybridization-based post-synthetic modifications, ligation reactions offer permanent attachment. The identity of *in situ*-synthesized DNA can be selectively transformed using a splint specific to the microarray feature of interest. However, a thorough investigation of the parameters governing on-array splint ligation is necessary, as surface confinement may alter reaction dynamics relative to solution-phase reactions due to differences in the accessibility of surface-immobilized probes.

In this work, we discuss key factors affecting splinted ligation on DNA arrays using the PBCV-1 (*Paramecium bursaria* chlorella virus 1) DNA ligase (commercially available from New England Biolabs as SplintR ligase). In particular, we examine parameters like phosphorylation methods, linker length, surface density, on-array sequence length, minimum effective splint length, probe mismatches, and the impact of repeated wash-off and ligation cycles on microarray-based RNA splint ligation. Additionally, we demonstrated applications of ligation, that includes low-background ssDNA probe labeling, on-array reverse transcription, and the incorporation of chemistries, such as photocleavable spacers, for spatial photopatterning.

## Methods

### Silanization of the Glass Surface

Microarray synthesis was performed on surface-functionalized glass slides as described previously.^37^ Briefly, 32.5 mmol of 3-aminopropyl triethoxysilane (Thermo Fisher Scientific) was used to functionalize Schott Nexterion D 263 glass slides (Schott 1095568) arranged in a stainless-steel rack (Sigma-Aldrich Z710989). The silanization mixture was prepared by mixing silane with ethanol in deionized water (95:5 v/v) and 0.2% acetic acid. The glass slides were kept in this mixture at room temperature with gentle shaking for 4 h. Then, the slides were washed twice for 20 min with a similar solution lacking the silane. The slides were then dried in argon and left in a 120 °C oven for the next 2 h under vacuum. After 2 h, the slides were left to cool down overnight under vacuum. Functionalized slides were stored in a desiccator until use.

### Microarray Design and Synthesis

All microarrays were synthesized using a maskless microarray synthesizer (MAS 2.0), which uses virtual masks displayed on a 1080p digital micromirror device (DMD) to photolithographically pattern high-density DNA microarrays on glass slides.^38–40^ The arrays have dimensions of 1920 × 1080 (2,073,600) with a feature pitch of 10.8 μm. All sequences in this study were designed to have ten thymidine linkers from the surface, except for linker gradient tests. For the acceptor arrays, sequences were synthesized with their 5′ termini bound to the surface using phosphoramidites carrying the 3′-BzNPPOC (benzoyl-2-(2-nitrophenyl)-propoxycarbonyl) groups (Orgentis),^41^ and for the donor arrays, sequences were immobilized on the surface via their 3′ termini using 5′-BzNPPOC phosphoramidites (Orgentis).^42^ These photolabile groups were removed using 9 J/cm^2^ and 4.5 J/cm^2^ of 365 nm light from a high-power UV LED,^43^ respectively, in the presence of 1% imidazole in dimethyl sulfoxide as exposure solvent.^44^ After synthesis, arrays were deprotected in 1:1 (v/v) ethylenediamine/ethanol (EDA/EtOH) at room temperature for 2 or 16 h for arrays containing 5′-BzNPPOC or 3′-BzNPPOC groups, respectively. Arrays were washed twice with distilled water and dried using a microarray centrifuge. All sequences used in this study are listed in the Supplementary Table S1.

### Chemical and Enzymatic Phosphorylation

Chemical phosphorylation of the test sequences at the 5′ termini was performed by using bis-cyanoethyl-*N*,*N*-diisopropyl CED phosphoramidites (ChemGenes) under standard synthetic conditions. For enzymatic phosphorylation using T4 polynucleotide kinase (T4 PNK), the terminal 5′ termini were exposed to UV in the final synthesis cycle to remove the photolabile group at selected positions. Negative control sequences that are not intended to have 5′ hydroxyl ends were capped using DMT-dT phosphoramidites (Glen Research). For enzymatic phosphorylation, the 1× T4 polynucleotide kinase reaction buffer was mixed with 1 mM ATP, 10 units of T4 PNK enzyme (all from New England Biolabs), and nuclease-free water. Microarrays were incubated with this mixture at 37 °C for 1 h to enzymatically phosphorylate the 5′ terminus. Before performing the ligation reaction, the reaction mixture was removed, and arrays were pipette-washed twice with 1× SplintR ligase reaction buffer. Splint ligation assays were performed either directly on arrays that were pre-phosphorylated or by introducing synthetic oligos chemically modified with 5′ terminal phosphate to an acceptor array of oligonucleotides with free terminal 3′ hydroxyls.

### Ligation Using SplintR Ligase

For RNA-splinted ligation on donor arrays modified with 5′-phosphate groups, 89 nM each of the RNA splint and Cy3-labeled acceptor oligo with 3′-terminal hydroxyl group (or donor oligonucleotides with 5′-phosphate in the case of acceptor arrays modified with 3′-terminal hydroxyl groups) was mixed with 1× SplintR ligase reaction buffer, 100 nM SplintR ligase, and introduced onto the array using a hybridization chamber (Grace Biolabs SA200) and incubated for 45 min in a hybridization oven with rotation at 37 °C. After the reaction, the solution was removed, and the arrays were washed gently with a nonstringent wash buffer (6× SSPE (Invitrogen), 0.01% Tween 20 (Carl Roth)) for 2 min, a stringent wash buffer (100 mM MES, 0.1 M Na^+^, 0.01% Tween 20) for 1 min, and a final wash buffer (0.1× SSC, Invitrogen) for 10 s, all in 50 mL Falcon tubes. The arrays were dried in a microarray centrifuge and scanned for fluorescence using a microarray scanner (GenePix 4400A, Molecular Devices) at 100% laser power, a PMT voltage of 350 V, an excitation wavelength of 532 nm, and at a resolution of 5 μm. To measure relative ligation efficiency, the arrays were treated with 50 U/mL RNase H and 1× RNase H buffer (New England Biolabs) for 1 h at 37 °C after ligation. The solution was removed after 1 h, and the arrays were washed again with the nonstringent wash buffer (2 min), a stringent wash buffer (1 min), and the final wash buffer (10 s). The arrays were dried, and scans were recorded as described before.

### Effect of Multiple Ligation Cycles

Acceptor sequences were synthesized on highly chemically resistant 3D-amino polymer slides (PolyAn). Donor sequences terminating with a 3′ poly­(dT) were ligated to the array as described previously. A pre-synthesized control sequence terminating with a 3′ poly­(dT) was incorporated into the workflow to quantify relative ligation efficiency. After each ligation cycle, the RNA splint was removed by incubating the array in a pre-warmed formamide buffer containing 50% (v/v) formamide (Sigma-Aldrich) and 2× SSC (Invitrogen) for 15 min at 55 °C. The stringency of the formamide wash was tested in parallel by measuring the loss of signal from a Cy3-labeled RNA splint. The arrays were washed with the routine wash buffers described previously, dried, and then scanned. Arrays were then hybridized with a Cy3-labeled complementary strand at 37 °C for 30 min using a 240 μL hybridization mixture that has 120 μL 2× MES buffer, 5.3 μL 20 mg/mL BSA, and 89 nM Cy3-labeled poly­(dA) handle. The previously described routine wash buffers were used to clean the hybridized arrays. After drying, the arrays were scanned to assess relative ligation efficiency. Prior to the subsequent ligation cycle, hybridized Cy3-labeled poly­(dA) oligos were removed from the array using formamide buffer, and any residual signal was documented. A total of four ligation cycles were performed, and each time, the intensity of the labeled poly­(dA) oligo from the ligated sequences was compared with that of the signal from the control probes on the same array.

### Biotin–Streptavidin-Based End Labeling

Biotinylated microarrays were synthesized as described above. The biotin was added via a coupling with 50 mM of 1-dimethoxytrityloxy-2-(*N*-biotinyl-4-aminobutyl)-propyl-3-*O*-(2-cyanoethyl)-(*N*,*N*-diisopropyl)­phosphoramidite (Glen Research) solution in dry acetonitrile. The standard coupling time for DNA (15 s) was also used for the biotin phosphoramidite. The biotinylated arrays were deprotected as usual, washed twice in distilled water, and dried. Arrays were first blocked with 2.8 μL of 20 mg/mL BSA (Invitrogen) in 1× PBS (Qiagen), diluted to 280 μL, and incubated at 25 °C with rotation in a hybridization oven. For biotin–streptavidin complex formation, a 280 μL reaction mixture composed of 100 nM Cy3-labeled streptavidin (Sigma-Aldrich), 1× PBS, and nuclease-free water was prepared. The blocking mixture was discarded and replaced with the reaction mixture, and the arrays were incubated at 25 °C for 3 h to form the biotin–streptavidin complex. The arrays were then briefly washed with a 0.1× SSC wash buffer for 10 s, dried, and scanned.

### Reverse Transcription on Ligated Sequences

Microarrays were designed to carry 5-nt-short acceptor sequences modified with free 3′-hydroxyl termini, along with pre-synthesized control T18VN sequences. Initially, ligation reactions were performed as described above, with 5′-phosphate-modified donor sequences at one end and a T18VN at the other. For this, the RNA splint and the donor sequences were mixed in an equimolar ratio (89 nM) in 1× splint reaction buffer, with 100 nM SplintR ligase, and nuclease-free water. The arrays were incubated in this mixture for 45 min at 25 °C with rotation. After this, the solution was removed, and the surface was pipette-washed twice with nonstringent wash buffer and once with stringent wash buffer. Washed arrays were refilled with 50 U/mL RNase H and 1× RNase H buffer, along with nuclease-free water. RNA-splint degradation was performed at 37 °C for 1 h. After this, the RNase H was heat-inactivated at 65 °C for an hour. The solution was removed and washed with the nonstringent wash buffer (2 min), followed by the stringent wash buffer (1 min), and a brief 10-s wash in the final wash buffer.

For mRNA capture, a 165 μL reaction mixture was prepared by mixing 6 μg universal human RNA (Agilent), 82.5 μL of 2× MES buffer (Sigma-Aldrich), and 3.65 μL of 20 mg/mL bovine serum albumin (Invitrogen), and brought to the final volume by addition of 75.2 μL water. The arrays with the mixture were incubated at 37 °C for 1 h. After RNA capture, the mixture was removed and briefly pipette-washed with the final wash buffer.

For reverse transcription, a published spatial transcriptomics protocol was adapted.^45^ Briefly, for a final volume of 120 μL reaction mixture, 12 μL of 10× M-MuLV buffer (New England Biolabs), 12 μL of 500 ng/μL of actinomycin D (Sigma-Aldrich), 1.124 μL of 20 mg/mL bovine serum albumin (BSA; Invitrogen), 6.1 μL of dNTP mixture (100 mM each of dATP, dTTP, dGTP and 25 mM of dCTP, Thermo Fisher), 3 μL of 0.2 mM Cy3-dCTP (Sigma-Aldrich), 6 μL of 40 U/μL of RNase OUT, 12 μL of 200 U/μL M-MuLV reverse transcriptase (New England Biolabs) and 67.8 μL of nuclease free water was mixed in the mentioned order, introduced onto the array and incubated at 42 °C overnight. After 16 h, the reverse transcription mixture was removed, and the arrays were washed with nonstringent and stringent wash buffers for two and 1 min, respectively, followed by a 10 s wash with the final wash buffer. Arrays were dried using a microarray centrifuge before scanning. Only successful reverse transcription provided a measurable signal.

### Data Extraction and Analysis

Reports containing probe intensities were extracted from the scan images using NimbleScan 2.1 (Roche-NimbleGen). The raw fluorescence signal intensities were first background-subtracted using the average signal from randomized background controls. All ligation efficiency results are based on fluorescence-based methods and are therefore subject to fluorescence-labeling artifacts. Particularly, the intensity of Cy3 fluorescence in a DNA microarray context depends on the sequence context as well as the length of the oligonucleotide separating it from the glass surface. These two effects have been accounted for by appropriate controls. Nevertheless, absolute reaction efficiencies generally cannot be measured on microarrays since multiple process efficiencies contribute to the signal. Therefore, ligation efficiency is represented as “relative ligation efficiency” and includes up to three required processes: phosphorylation efficiency, splint hybridization efficiency, and ligation efficiency. In the case of acceptor arrays, where the 5′-phosphate-modified purified donor sequences are added, only hybridization and ligation efficiency contribute to the relative ligation efficiency. To calculate relative ligation efficiency (*RLE*), intensities after RNase H treatment or formaldehyde wash treatment (*I*_after_) were normalized to the mean hybridization signal of a reference probe (*I*_reference_) on the same array, *RLE*_*i*_ = (*I*_after *i*_ – <*I*_background_>)/(<*I*_reference_ – <*I*_background_≫) × 100; here, the subscript *i* is an index over the replicates and <*I*_background_> is the mean of *i* background features on each array. In Figure 3, due to the absence of an equivalent reference probe across different sequence lengths, ligation efficiency is represented as the retention signal (*RS*). The retention signal was calculated as the ratio of the Cy3 fluorescence intensity from the 5′-end-labeled acceptor probe after RNase H (*I*_after_) treatment to that before RNase H treatment (*I*_before_), *RS*_*i*_ = (*I*_after *i*_ – <*I*_background_>)/(*I*_before *i*_ – <*I*_background_>).

## Results and Discussion

### Chemical vs Enzymatic Phosphorylation

We first attempted different phosphorylation strategies to make a 5′-phosphate donor on the array. Both enzymatic and chemical phosphorylation were performed as described above. The general scheme for on-array ligation is shown in Figure 1a. For enzymatic phosphorylation, oligonucleotides with the 5′-terminal hydroxyl group will, after T4 PNK treatment, be the phosphate-bearing donors (Donor (+)). Oligonucleotides not terminating with the free 5′-terminal hydroxyl group will not be a substrate for T4 PNK (Donor (−)). Similarly, only Donor (+) strands will be phosphorylated after chemical phosphorylation using maskless photolithography. In both cases, sequences were designed to carry 20 bases complementary to the RNA-splint from both the acceptor and donor ends, as shown in the scheme.

**Figure 1:**
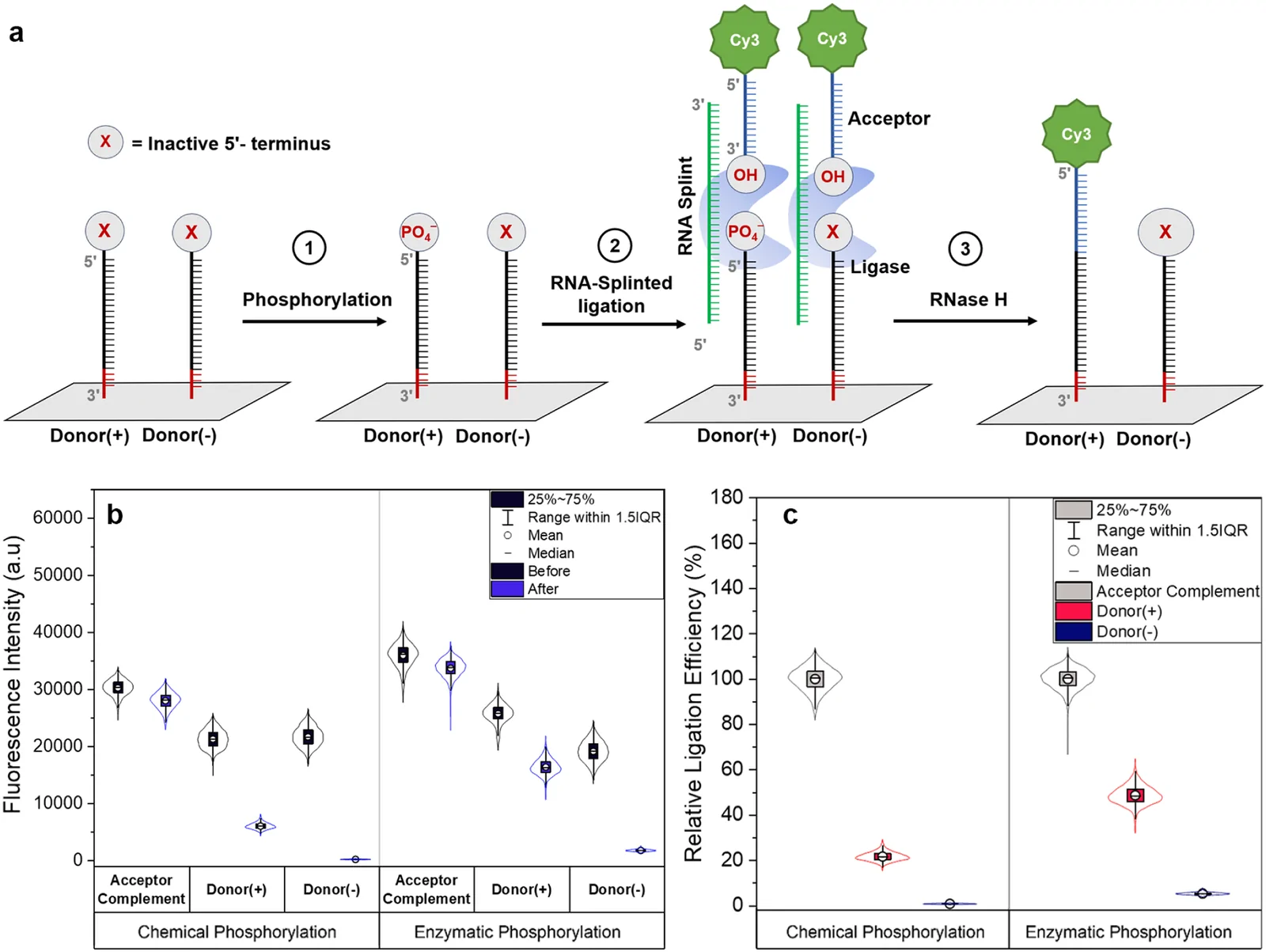
**a**. Scheme showing the on-array splint ligation protocol: (1) After photolithographic synthesis, sequences on the array are phosphorylated using either chemical or enzymatic phosphorylation. X represents the inactive 5′ terminus for phosphorylation. (2) Splinted ligation was performed by SplintR ligase in the presence of a Cy3-labeled acceptor strand with a terminal 3′-hydroxyl group and an RNA splint complementary to both the donor and acceptor strands. (3) After RNase H treatment, the signal is retained only on the phosphorylated Donor (+) strand. **b**. Fluorescence intensity of the sequences before and after RNase H treatment. **c**. Relative ligation efficiency of both chemical and enzymatic phosphorylation was calculated by normalizing signals measured after RNase H treatment with respect to a reference probe complementary to the Cy3-labeled acceptor sequence. Data for each experimental condition are combined from two replicate arrays, each representing the signal from 3,834 on-array replicates.

The raw intensities measured before and after RNase H treatment for Donor (+) and Donor (−) strands are shown in Figure 1b. The performance of both ligation approaches was evaluated by measuring the signal after RNase H treatment in the Donor (+) and Donor (−) sequences and normalizing to the hybridization signal from a reference probe complementary to the incoming acceptor oligonucleotide (Figure 1c). Ligation of strands that were chemically or enzymatically phosphorylated achieved relative ligation efficiencies of 21.7 ± 0.2% and 48.7 ± 2.4% (mean ± SD), respectively, under equivalent ligation conditions from two array replicates per phosphorylation condition, each carrying 3834 on-array replicates. Therefore, we employed an enzymatic phosphorylation strategy for further assays. In our initial experiments, Donor (−) strands were capped with 5′-BzNPPOC protection groups instead of DMT-based protection, but were observed to have unintended ligation even in the absence of active 5′ termini. This effect was not observed in the absence of ligase (Supplementary Figure S1) or with chemical phosphorylation (data not shown).

A similar behavior was observed in experiments in which ligation was performed in an acceptor array with sequences terminating in 3′-BzNPPOC groups, in which externally introduced 5′-phosphorylated strands facilitated ligation (data not shown). Hence, we hypothesized that under standard basic deprotection conditions (1:1 (v/v) EDA/EtOH; ≥2 h), used to remove backbone protection groups, BzNPPOC groups are partially cleaved from the DNA strands and generate free 5′- or 3′-hydroxyl termini, which could subsequently be phosphorylated by T4 PNK treatment or through the introduction of pre-phosphorylated donor strands. This effect is not expected in chemical phosphorylation, as maskless microarray synthesis offers spatial control, ensuring that only intended oligonucleotides are phosphorylated. Hence, for site-selective phosphorylation, either chemical phosphorylation or the use of non-base-labile protecting groups offers a reliable strategy.

### Strategies to Improve On-Array Ligation Efficiency

Given the moderate relative ligation efficiency on microarrays, we explored several strategies to improve performance. Initially, we hypothesized that increasing the distance between the substrate oligonucleotides and the surface by adding longer linkers might improve array ligation. Consequently, donor strands were synthesized with thymidine linkers of varying length (T1-T15). There was no dramatic increase in the relative ligation efficiency with increasing linker length (Supplementary Figure S2). Next, we attempted to vary incubation time and ligase concentration, but neither resulted in a significant improvement (Supplementary Figure S2). We also tried preannealing the RNA splint with the acceptor before ligation, but the effect was the same. Furthermore, reducing the effective concentration of the splint or the acceptor probes decreased relative ligation efficiency (data not shown).

We then tried multiple ligation cycles on the same array (Figure 2a). We have previously found that the yield of on-array psoralen-based cross-linking reactions can be significantly increased via consecutive attempts.^8^ For this, we used the much more stable surface functionalization offered by 3D amino slides to better withstand the increased handling. The 3D amino-functionalized surfaces ensured that surface-bound probes stay intact during four ligation cycles and corresponding wash steps. Donor sequences terminating with a 3′-poly­(dT) end were introduced by ligation. After each ligation cycle, a formamide wash removed the RNA splint, leaving only the ligated sequences bound to the array. A photolithographically synthesized sequence terminating in 3′-poly­(dT), with a sequence context similar to that of the ligated one, served as a reference probe. A control sequence with an inactive 3′ terminus was also added. All sequences were hybridized to a Cy3-labeled poly­(A) oligonucleotide to measure relative ligation efficiency. Before moving to the next round of ligation, residual signals from Cy3-labeled polyadenylated sequences were removed by formamide wash. Four ligation cycles were carried out, each lasting 45 min.

**Figure 2:**
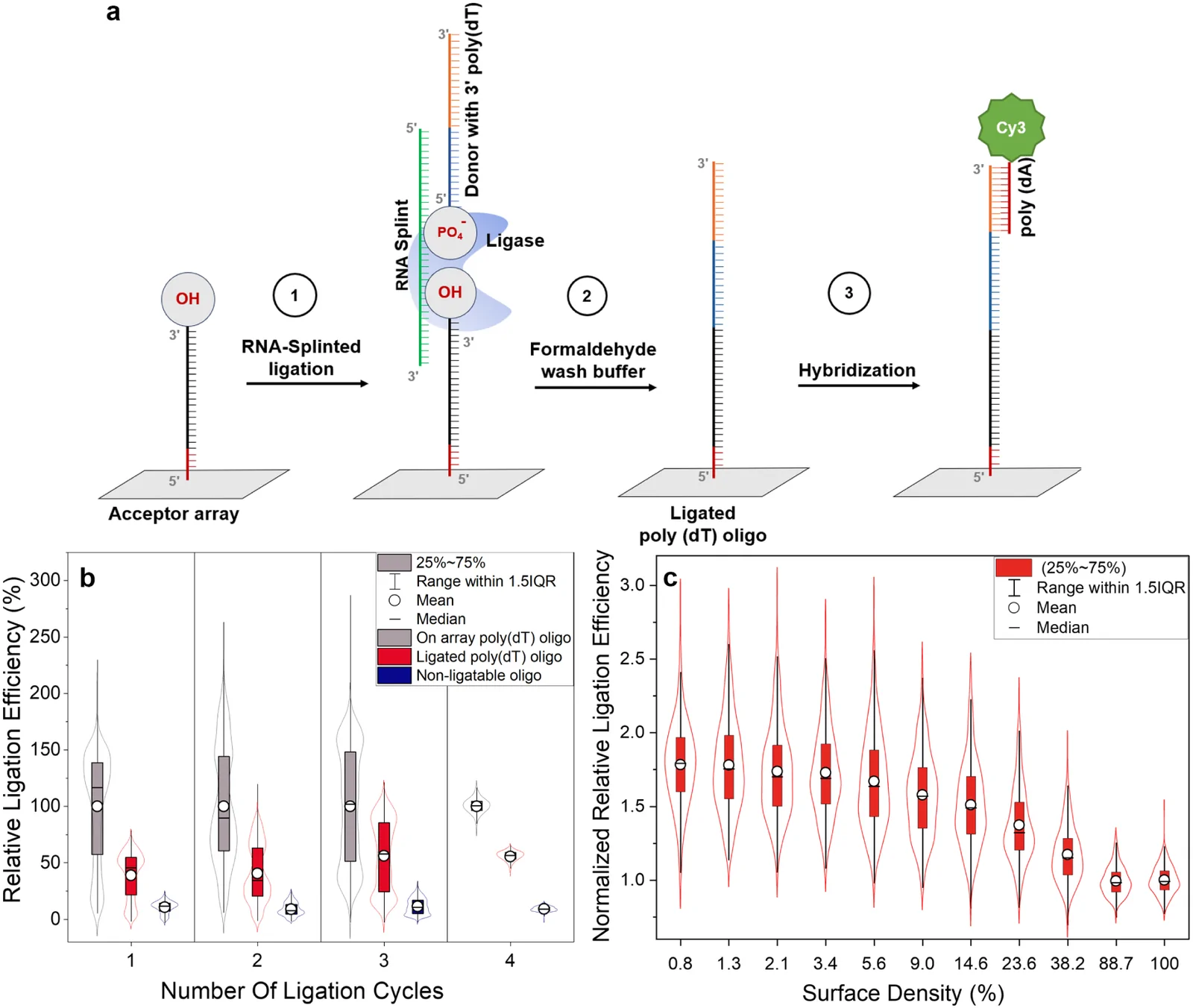
**a**. Scheme showing the experimental workflow used for measuring the effect of multiple ligation cycles. **b.** Signal intensities of sequences under different ligation cycles were normalized to the corresponding on-array poly­(dT) oligo. Data from a single array and 3833 on-array replicates. **c.** Effect of surface density of oligonucleotides on relative ligation efficiency. Data from a single array and 276 on-array replicates, normalized to full density (100%).

Following the third ligation cycle, the reaction reached a plateau at approximately 56% relative ligation efficiency (Figure 2b). 5′-adenylated DNA ends can act as trapped intermediates that stall ligation when the enzyme is readenylated before nick sealing. However, adenylation is transferred exclusively onto the 5′-phosphate, which in our design is supplied by the donor strand, whereas the immobilized acceptor strand presents only a 3′-hydroxyl and therefore cannot form such an intermediate. Because each cycle introduces fresh 5′-phosphorylated donor strands, ligase, splint, and buffer following a stringent wash, any adenylated donor that fails to ligate is removed with the spent reaction rather than carried forward. Progressive accumulation of dead-end intermediates is therefore unlikely to account for the observed plateau.

Given that the ligation reaction plateaus after the third cycle, we wondered whether the high oligonucleotide surface density partially impedes ligation. We have noticed similar effects in other on-array enzymatic reactions where effective enzyme efficiency has been moderate.^15,16^ To investigate this, we introduced an oligonucleotide surface density gradient via a UV exposure gradient on the final nucleotide of the T linker. Following the exposure gradient, the free hydroxyls were capped with DMT-dT, since the DMT caps oligonucleotide extension in the absence of an acidic deblocking step in photolithographic synthesis. Microarray features with zero UV exposure before DMT capping exhibit a high surface density, whereas fully exposed features exhibit a greatly reduced surface density (Supplementary Table S2). Reference sequences complementary to the Cy3-labeled acceptor sequences were also synthesized in parallel, so that each functions as a control for its respective exposure.

Data from the ligation reaction on this array show that reducing surface density decreases the absolute signal intensity but preserves higher relative signal intensity compared with the respective reference probes (Figure 2c, Supplementary Figure S2). In summary, reducing the surface density significantly increased relative ligation efficiency but at the significant expense of lower overall signal, due to fewer oligonucleotides per feature.

### Effect of On-Array Oligonucleotide Length

Next, we tested the effect of on-array sequence length for ligation. For this, we designed sequences with minimal secondary structures using NUPACK^46,47^ and truncated them from their 3′ end, each with a 10 bp difference and varying in length from 15 nt to 115 nt. All sequences had at least 15 nt of complementary base-pairing with the RNA splint and a 10-base-pair thymidine linker on the surface. A 22 bp Cy3-labeled acceptor strand was introduced onto the array, along with a 40 bp RNA splint. Since each sequence on the array had varying lengths, the relative ligation efficiency is represented as a normalized retention signal and was calculated as the ratio of Cy3 signals before and after RNase H treatment. According to our data, changes in sequence length do not greatly impact the retention signal (relative ligation efficiency). This suggests that SplintR ligase reliably identifies the 5′ phosphate and 3′ hydroxyl ends when an RNA splint is present, efficiently joining strands even when their sequence lengths differ (Figure 3a). Raw fluorescence signals before and after RNase H treatment are shown in Supplementary Figure S3.

**Figure 3:**
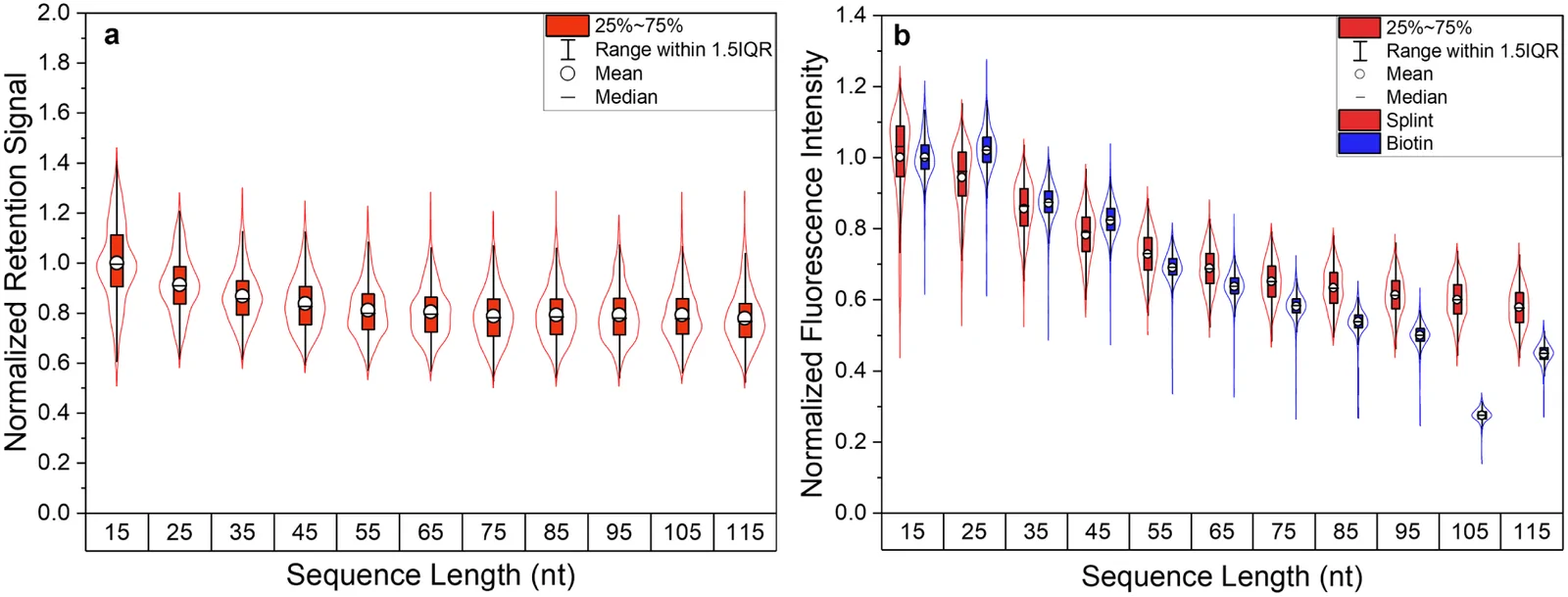
**a**. Normalized retention signal is calculated as a ratio of after and before RNase H treatment on varying sequence lengths and was largely unaffected by sequence length, whereas **b.** Fluorescence signal from terminal labeling using biotin or splint ligation on different sequence lengths was affected by sequence length. Data for each condition were generated from single arrays in each case and 1045 on-array replicates.

Direct labeling with Cy3 phosphoramidites was previously observed to yield very high background signals due to strong binding of the Cy3 phosphoramidite to the glass substrate.^13^ Ligation provides an alternative strategy for labeling or otherwise modifying sequences after synthesis. Alternatively, biotin-based end labeling followed by Cy3-labeled streptavidin treatment also offers terminal Cy3-labeled sequences with minimal background noise. Hence, we compared ligation-based terminal labeling of ssDNA sequences with Cy3 to biotin–streptavidin-based end labeling. For this, sequences were designed in NUPACK with similar truncations and coupled biotin phosphoramidites to the 5′ termini. Successful biotin–streptavidin complex formation generates a measurable fluorescence signal. Both end-labeling approaches were nearly comparable, and Cy3 signals were highly dependent on sequence length in both cases (Figure 3b). We attribute the length dependence to the proximity of the Cy3 to the surface. Surface-proximal Cy3 is more likely to interact with the glass surface, thereby enhancing fluorescence by restricting rotational isomerization.^12,13^ Taken together, the reduction of fluorescence signals with increasing sequence length is primarily attributed to Cy3-related artifacts, whereas the relative ligation efficiency is largely unaffected.

### Effect of RNA Splint Length

During DNA synthesis, error rates per oligonucleotide increase with the number of synthesis cycles because the stepwise phosphoramidite coupling efficiency is under 100% (typically 98–99.9%).^41,44^ Therefore, in specific applications where higher-fidelity, longer oligonucleotides are required, and these oligonucleotides share a common terminal sequence, combined *in situ* synthesis followed by ligation of a shared subsequence is advantageous. However, the need for significant complementary base pairing between the donor or acceptor and the splint RNA will offset the increased synthesis efficiency. This complementarity has been studied in solution-based assays.^33^ For complex arrays of surface-bound oligos, no equivalent information is available. To check the minimum length of the RNA splint that results in successful ligation, we evaluated three different splint lengths, 20 nt, 30 nt, and 40 nt, such that both the donor side and acceptor side had the same number of base pairs with the RNA splint (Figure 4a). We observed that a 20 nt splint with 10 nt complementary base pairing on both the acceptor and donor sides was sufficient to achieve ligation (Figure 4b).

**Figure 4:**
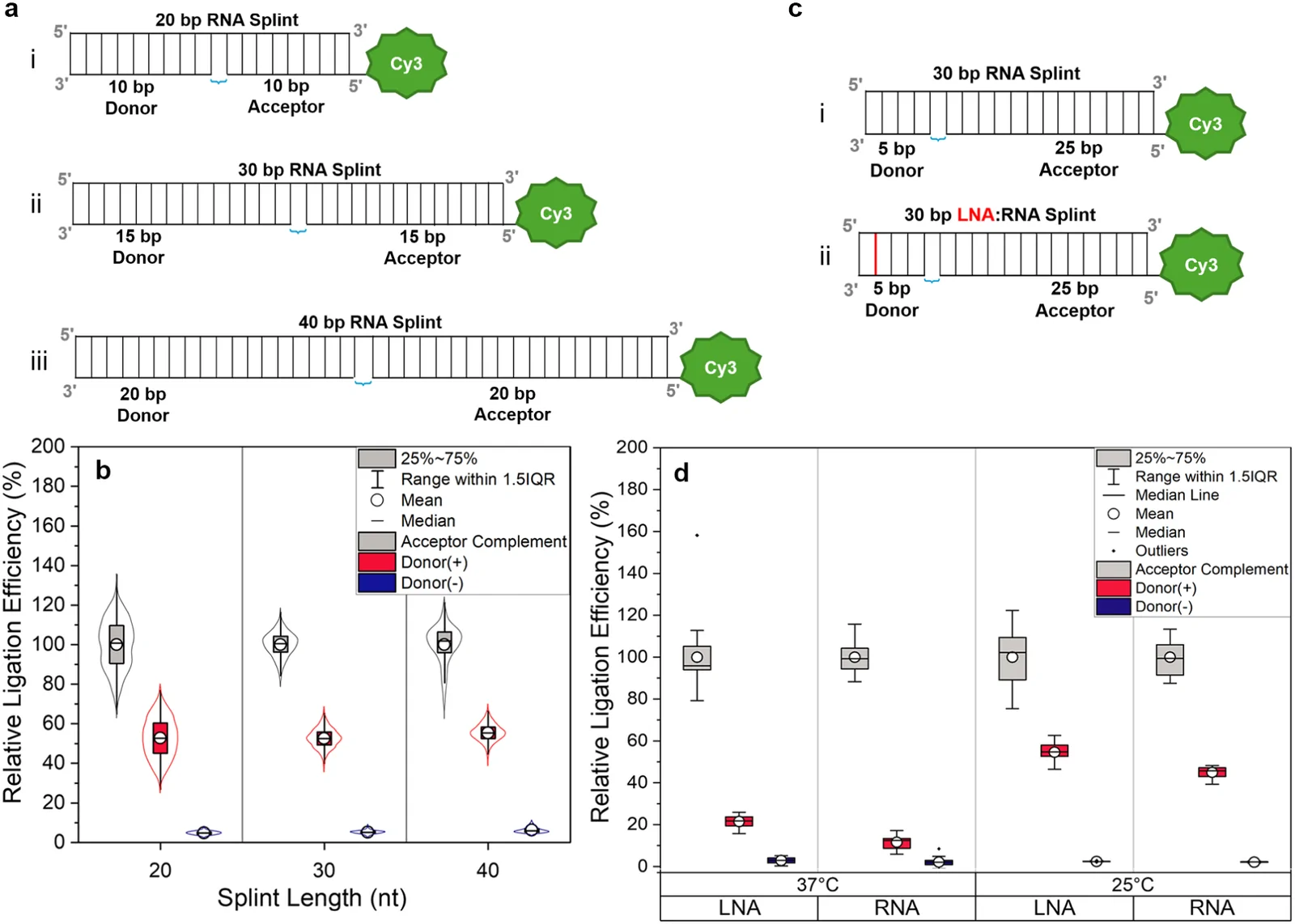
**a.** Scheme showing splint RNAs that are (i) 20 bp, (ii) 30 bp, and (iii) 40 bp long and have the same base-pairing lengths on either side of the ligation junction (blue bracket), and **b.** Represents the corresponding relative ligation efficiency (red). Normalization was performed with respect to a reference probe complementary to the Cy3-labeled acceptor sequence. Data from a single array and 2654 on-array replicates. **c.** (i) Represents the 30 bp RNA-only splint hybridized to short 5 bp donor and 25 bp acceptor sequences (ii) RNA residue at the fourth position replaced by LNA (red). **d.** Relative ligation efficiency of the sequence with moderate GC content at 25 and 37 °C. Data from a single array and 17 on-array replicates.

Next, we attempted to ligate a 5-nt-long donor strand to a 25-nt-long Cy3-labeled acceptor strand using a 30-nt RNA. We hypothesized that, with sufficient duplex stability on the acceptor side, it may be possible to efficiently ligate with only 5-nt donor-splint complementarity. Since a 5 nt donor-RNA duplex close to the ligation junction may not be sufficiently stable, we replaced a single RNA base at the fourth base-position away from the junction with an LNA nucleotide (Figure 4c). In parallel, we tested donor sequences with high GC content (60%), moderate GC content (40%), and low GC content (0%) at ligation temperatures of 25 and 37 °C, keeping the −1 and −2 bases at the ligation junction constant. Relative ligation efficiency for extreme GC sequences (low GC and high GC), and raw fluorescence intensities of all sequences tested are shown in Supplementary Figure S4. Relative ligation efficiency for moderate GC sequences is shown in Figure 4d.

Across all GC ranges, relative ligation efficiency was lower at 37 °C than at 25 °C. Low GC sequences were not rescued by a single LNA base at 37 °C, but were slightly improved at 25 °C. Moderate GC sequences showed reasonable performance with both the RNA-only splint and the LNA-splint at both temperatures.

The high GC RNA-only splint performed moderately, but the LNA-containing splint outperformed it at both temperatures; at 25 °C, we observed a 35-fold improvement over the reference probe. Surprisingly, from the unnormalized fluorescence signal, we observed that for the LNA-containing splint, the raw signal from the reference probe was much lower than that from the high-GC sequence at both temperatures. This effect was not observed with normal RNA (Supplementary Figure S4) or with other GC ranges tested. We believe this is likely an artifact due to the higher duplex stability of the RNA:DNA hybrid in the presence of a single LNA base, which becomes more pronounced at higher GC and lower temperatures, thereby capturing incoming acceptor sequences for hybrid formation and reducing the local concentration on the reference hybridization probe. We analyzed the normalized and raw signals and concluded that ligation can occur with a 5-nt donor, with better performance observed with sequences of moderate or higher GC content. LNA is not necessary at room temperature.

Even though we have not evaluated the performance of sequences with different GC ranges on the acceptor side of the ligation junction, we demonstrated that a 5-nt acceptor sequence with a moderate GC content, together with an RNA-only splint, can essentially be used for ligation when performed at 25 °C and can be further used for downstream enzymatic assays (Figure 6).

### Effect of Mismatches on Acceptor and Donor Strands

To study the tolerance of SplintR ligase to splint mismatches on the surface-bound oligos, we generated all possible single and double mismatches on both an acceptor array with terminal 3′-hydroxyl groups and a donor array with 5′-phosphate termini (Figure 5a). In each case, the second strand with an active terminus was externally introduced, together with the RNA splint. Our findings from the most frequently ligated sequences indicate that distal modifications on both sides of the junction did not affect ligation. On the acceptor side, distal modifications that are 5 bp away from the junction were tolerated (Figure 5b). Similarly, on the donor side, distal modifications 7 bp away from the junction could ligate to the acceptor strand (Figure 5c). On the other hand, the least ligated sequences were enriched with mismatches close to the ligation junction. On the acceptor side, the −1 position was more sensitive compared to neighboring positions (Figure 5d), whereas on the donor side, ligation was sensitive to any modifications at both +1 and +2 positions (Figure 5e; Supplementary Table S3).

**Figure 5:**
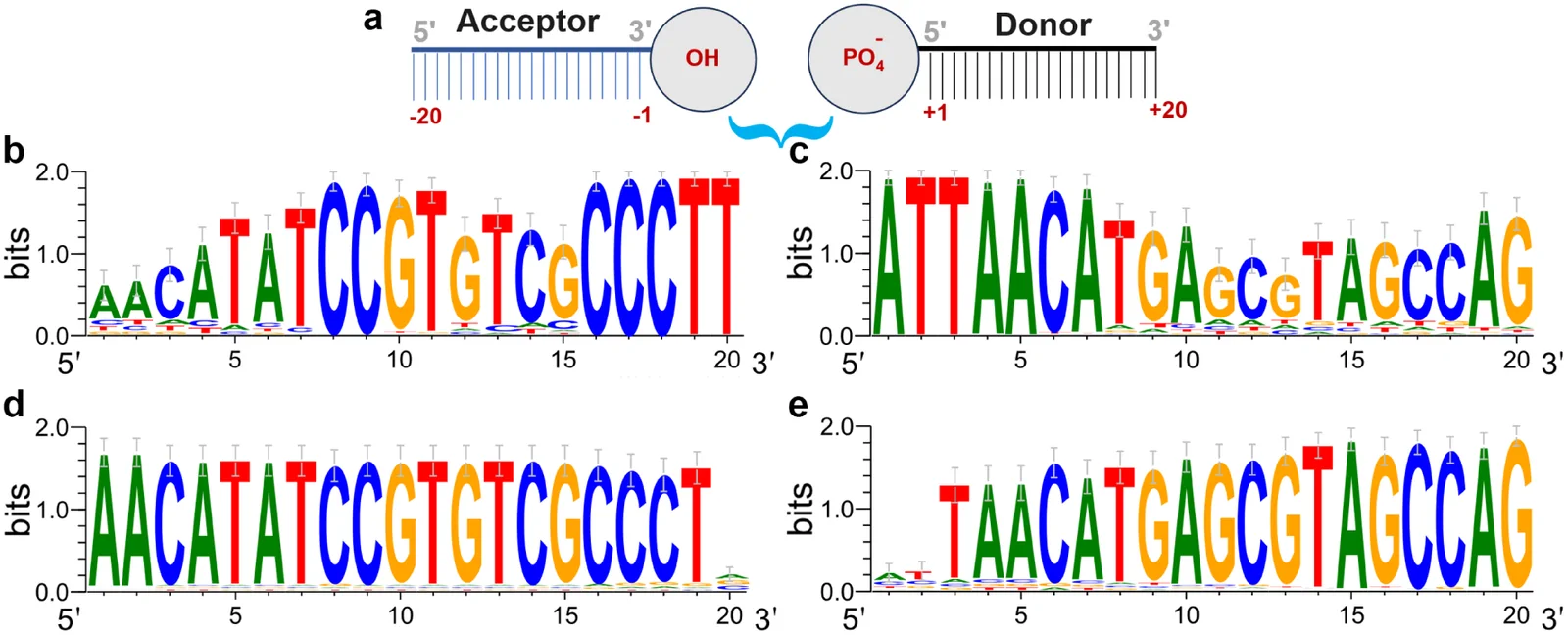
**a.** Scheme representing the ligation junction (blue bracket) and the alignment of the 20 bp acceptor and 20 bp donor sequences, along with the numbering logic used to indicate the positions. **b.** and **c.** Represent the WebLogo generated for highly ligated (10%) sequences on the acceptor side and donor side, respectively. **d**. and **e.** Show the least ligated (10%) sequences on the acceptor and donor sides, respectively. A sequence logo (or WebLogo) is a graphical representation of a set of aligned DNA sequences, where each position shows a stack of letters whose total height reflects how conserved that position is, and each letter’s height reflects its relative frequency.

### Reverse Transcription Assays

It is also interesting to extend the ligation workflow to the efficient synthesis of arrays for spatial transcriptomics of plant and animal tissue slices.^45,48^ These arrays are typically made using spotting of premade oligonucleotides, but the technology would benefit from an efficient *in situ* synthesis-based manufacturing approach. The array oligonucleotides are attached to the surface at their 5′ ends and generally share a 5′ subsequence that includes an enzymatically cleavable linker (generally dU) and a sequencing handle. This is followed by a spatial barcode, a unique sequence of nucleotides that uniquely identifies the original position of the oligonucleotide on the surface in the sequencing data. The 3′ terminus features a Unique Molecular Identifier (UMI), a subsequence randomized at the molecular level using couplings from an equimolar mix of DNA phosphoramidites, and used to identify duplicate molecules produced during PCR, ensuring accurate quantitative gene expression counts. The UMI is followed by a poly­(dT) capture subsequence, a tail of deoxythymidines that hybridize to the polyadenylated (poly­(A)) tail of mRNA molecules diffusing from the tissue section. Following on-array synthesis of the 5′ subsequence, including the barcode, and then introducing the 3′ UMI and capture subsequences by ligation could reduce both synthesis errors and cost. Additionally, it would enable the partial substitution of LNA T, a chemistry not yet available for photolithographic synthesis, in the capture oligonucleotide. LNA is frequently used in poly­(dT) capture oligonucleotides to significantly increase hybridization affinity and thermal stability.^49,50^

As a proof-of-concept for functional use, mRNA capture sequences ligated to the microarray oligonucleotides were used in a reverse transcription assay. With sufficient duplex stability for the DNA-RNA splint hybrid at the ligation junction, a 5 nt sequence synthesized on the array was shown to be both sufficient and efficient for RNA-splinted ligation when performed at a lower temperature (Figure 4d). Hence, acceptor arrays were synthesized with a 5 bp sequence complementary to the 23 nt splint RNA. The 48 nt donor-side oligonucleotides, which consisted of the RNA splint complementary sequence, a 10 nt UMI sequence (N_10_), and the capture sequence T_18_VN, were first ligated to sequences on the acceptor oligonucleotide array with terminal 3′-hydroxyl groups. The VN terminus enforces alignment of the capture oligonucleotide with the poly­(A)-3′ UTR junction of the mRNA. Next, mRNA transcripts from total human RNA were captured via their poly­(A) tails and reverse transcribed. Successful reverse transcription yielded fluorescence signals from Cy3-labeled dCTP incorporated by the reverse transcriptase. A simple scheme is shown in Figure 6a. To compare the capture efficiency of the ligated N_10_T_18_VN capture sequences, acceptor arrays were prepared that also included photolithographically synthesized capture oligonucleotides as controls (Figure 6b). Fluorescence signals from the ligated oligonucleotides were normalized against signals from the *in situ* synthesized capture oligonucleotide controls. Signals were observed from both the ligated sequence and the on-array synthesized control oligonucleotides when ligase was present (Figure 6c). In contrast, in the absence of ligase, only the on-array-synthesized capture control oligonucleotides produced a Cy3 signal after reverse transcription. Overall, the on-array capture control oligonucleotide performs better than the ligated capture oligonucleotide, which exhibited a capture efficiency of ∼60%. The measured signal represents a cumulative outcome of the complete workflow, including ligation, mRNA capture, reverse transcription, and fluorescence detection. However, since the ligated capture probe was compared directly with the in situ T18VN capture probe present on the same array, which underwent the identical hybridization, reverse transcription, washing, and fluorescence detection procedures, the downstream capture and reverse transcription steps are controlled for within the experiment. Hence, under these conditions, the reduced signal observed for the ligated capture probe is therefore expected to arise predominantly from incomplete ligation, although we cannot completely exclude smaller contributions from differences in probe accessibility or local surface environment introduced by the ligation process.

**Figure 6:**
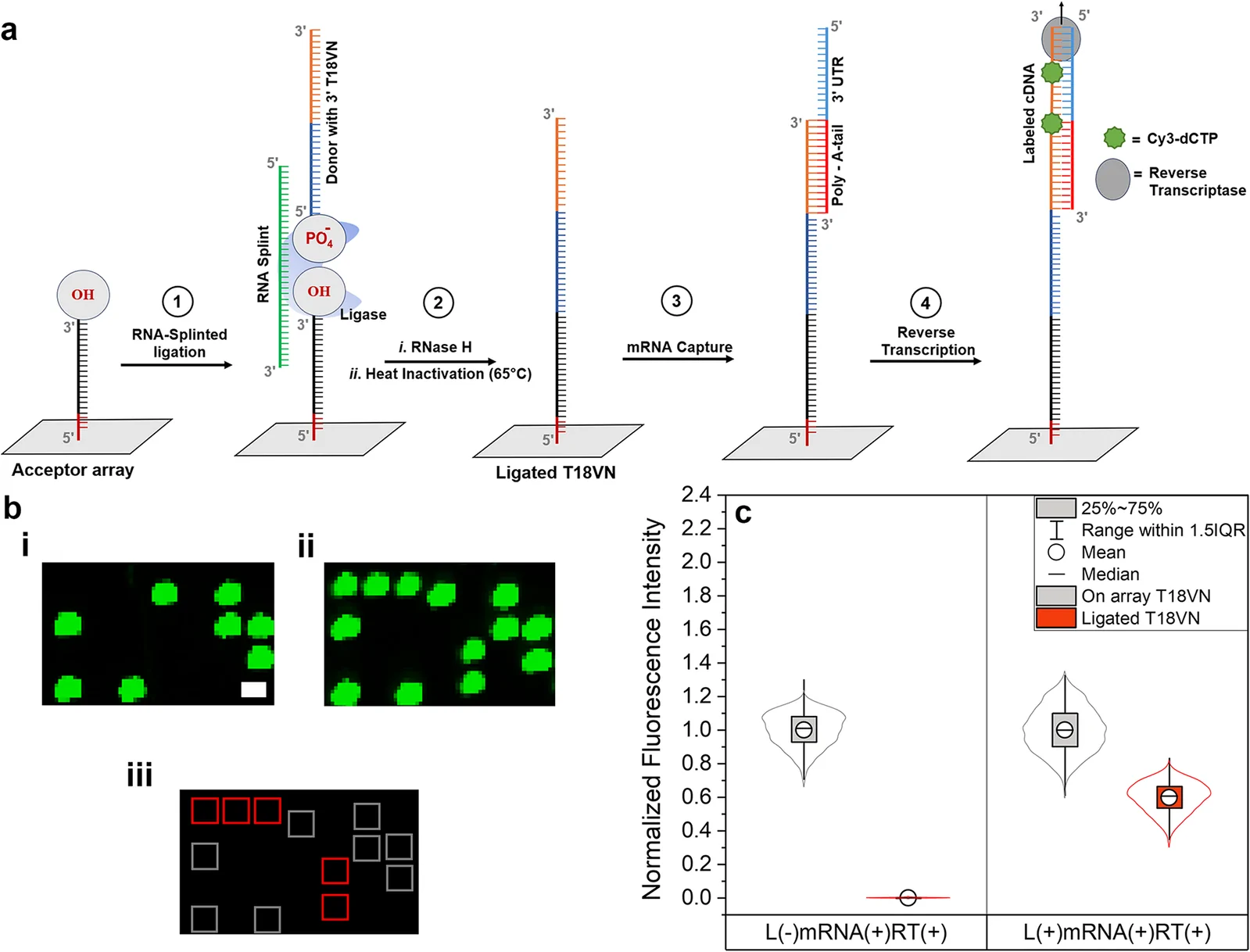
**a.** Scheme showing ligation-based workflow for reverse transcription on microarray. **b.** Excerpts of an array treated with reverse transcription mixture in the (i) absence of ligase and (ii) in the presence of ligase. Feature identity is represented in (iii) with gray squares corresponding to on-array T18VN and red squares populated with ligated T18VN sequences. Scale bar 10 μm. **c.** Normalized fluorescence intensity of two different sequences under two different conditions. L(−) mRNA­(+) RT­(+) represents an array where ligation was not performed before mRNA capture and reverse transcription. L­(+) mRNA­(+) RT­(+) corresponds to an array where ligation was performed before mRNA capture and reverse transcription assay. Data from a single array with 5750 on-array replicates.

### High Complexity Post-Synthetic Surface Modification Using RNA Splint Ligation

Our group has previously published multiple post-fabrication strategies for microarrays, primarily based on nucleic acid nucleases and polymerases. Ligation reactions are unique in allowing the addition of entire oligonucleotides to the *in situ*-synthesized microarray surface. Moreover, ligation reactions can be used to introduce versatile chemistries that are incompatible with light-directed microarray synthesis. An important group of such chemistries are UV-active modifications. Since photolabile protecting groups are used to control the synthesis, they cannot also be used as a modification in the final product. DNA-linked photocleavable groups are important tools for precisely controlling the behavior of molecules. They have found diverse applications, including DNA logic gates,^51,52^ surface patterning,^53–55^ cell-free genetic circuits^56,57^ and nanotechnology,^58–61^ among many others.^62,63^

To demonstrate the use of ligation to introduce a photocleavable spacer, as well as the control of light exposure that can be achieved with the MAS 2.0 maskless array synthesizer, we converted a grayscale photograph of chemist and crystallographer Rosalind Franklin (Figure 7a) into a fluorescent DNA microarray image by adapting the ligation workflow. First, we synthesized a single-sequence 5′-phosphorylated microarray, which was then ligated to a 5′-Cy3-labeled 3′-hydroxyl sequence internally modified with an *o*-nitrophenyl-based photocleavable spacer (PC spacer), a linker that cannot normally be used because it cleaves during synthesis (Figure 7b). To reproduce the image, it was first converted to 1080p (1920 × 1080) format, matching the dimensions of our digital micromirror device. The grayscale image′s bit depth was also reduced from 8 bits (256 shades of gray) to 38 shades of gray to simplify the process while maintaining high visual quality (Figure 7c). To account for our higher sensitivity to changes in the darker areas of an image, the exposure times corresponding to each shade of gray were chosen to be approximately logarithmic, with closer spacing between darker gray levels (Supplementary Table S4). The ligated array was reinserted into the MAS 2.0 optical synthesis for controlled cleavage of the PC spacer. The 38 virtual masks were then displayed sequentially, using an automated display script, to irradiate the surface. The exposures were performed in nuclease-free water, using a radiant intensity of 9 mW/cm^2^ at 365 nm (Figure 7d). Sequential exposure was controlled by the same software normally used to direct microarray synthesis. To calibrate the exposure necessary to cleave the PC spacer, an initial exposure gradient assay was performed on an equivalent ligated array. The exposure-dependent loss of fluorescence intensity was plotted as a function of exposure time. Data were fitted with a first-order kinetic equation, and the rate constant was extracted (*k* = 0.00735 s^–1^). This parameter was subsequently used to calculate the exposure time for each virtual mask required to recreate the image (Supplementary Figure S5). The resulting fluorescent scan and its grayscale representation (Figure 7e, f) closely reproduce the original image.

**Figure 7:**
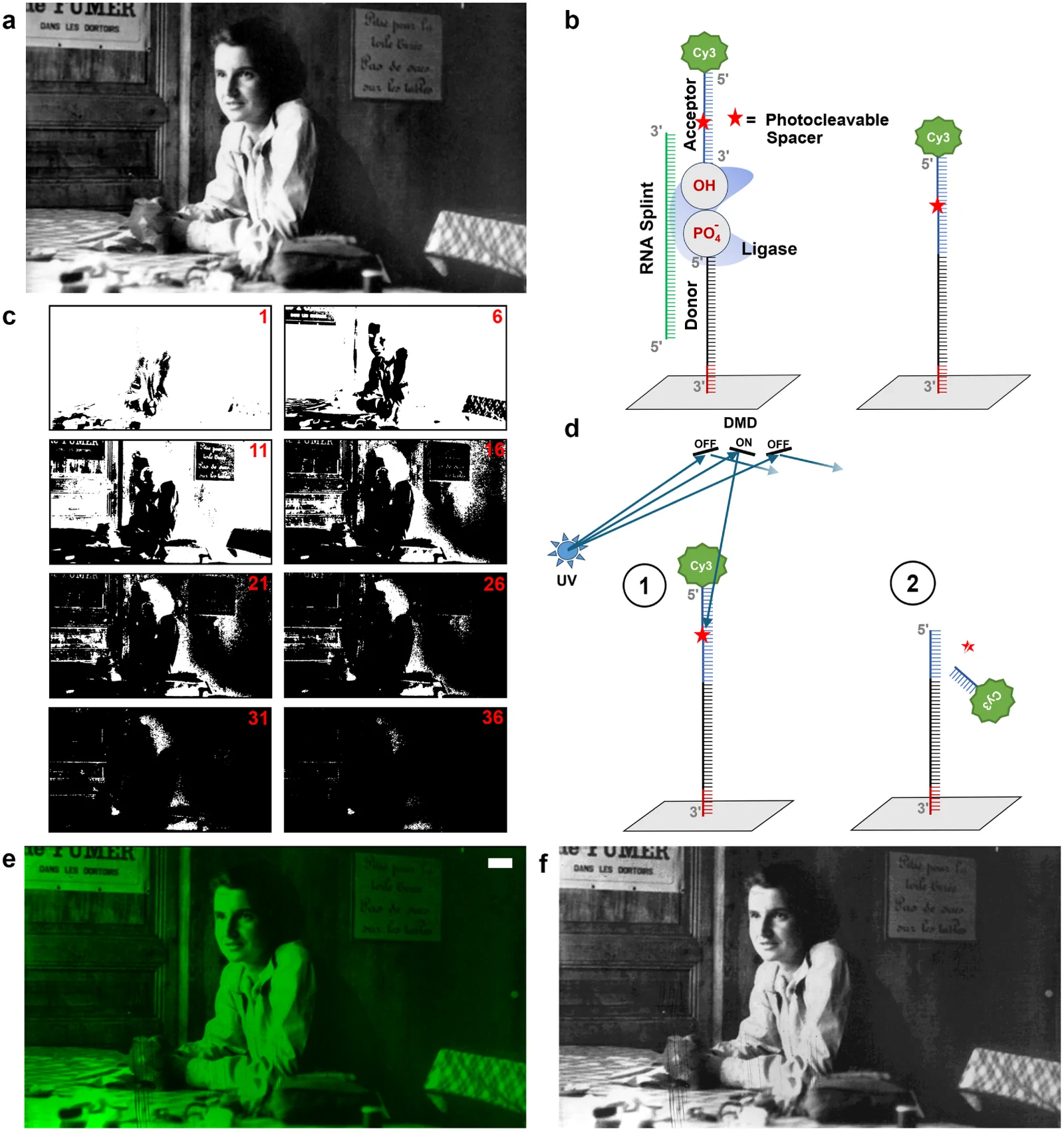
**a.** Grayscale image of Rosalind Franklin (Photo by Vittorio Luzzati; © National Portrait Gallery, London; reproduced with permission. This image is excluded from the CC-BY 4.0 license of this article). **b.** Prior to patterning, the full surface of the 1080p array is functionalized as a donor array and ligated with an oligonucleotide bearing an internal photocleavable spacer and a 5′ Cy3 (1), yielding an array of photoaddressable oligonucleotides (2). **c**. Selection of the 38 virtual masks used to create the exposure patterns to reproduce the original image in Cy3-labeled DNA. **d**. The ligated array is patterned by time-controlled exposures with the 38 virtual masks displayed consecutively on the DMD (1). The controlled UV exposure results in highly selective cleavage of the spacer (2). **e**. Fluorescence image of the resulting microarray, scale bar 1 mm. **f**. The final grayscale image on the microarray closely reproduces the original. The physical dimensions of the 1080p array in **e** and **f** are 20.74 × 11.67 mm.

## Conclusion

In this work, we adapted an RNA splint ligation strategy to high-density microarrays synthesized *in situ* by digital nucleic acid photolithography and quantified multiple factors that determine the efficiency of on-array ligation, as well as its potential for downstream applications. These factors are enzymatic vs chemical phosphorylation of the array donor oligonucleotides, linker length, oligonucleotide length, splint length, mismatches to the splint, cycles of ligation reactions, and oligonucleotide surface density. The primary finding is that SplintR ligation on the microarray surface is less efficient than in solution, in the range of 50 to 60%. By comparison, efficiencies of 70% and above have been reported in solution-based assays.^33,64^ These values are consistent with our surface assays in which the relative ligation efficiency can be increased to this range by greatly reducing the oligonucleotide surface density. This observation is consistent with previous measurements of reduced enzymatic activity in crowded nucleic acid environments in surface-based assays, which can be attributed to molecular crowding and steric interference by the substrate.^65–69^ Nevertheless, extension of surface-bound oligonucleotides via splint-assisted ligation is a useful tool for introducing modifications that cannot be introduced otherwise. These include long sequences that are time-consuming or expensive to introduce via phosphoramidite coupling reactions, or those that are not compatible with the photochemical synthesis. In addition, the approach enables adding more than one oligonucleotide per feature, for example, to simultaneously capture both eukaryotic and prokaryotic RNA in spatial transcriptomics. In all these contexts, the ligation reaction is straightforward and fast, and requires as little as five nucleotide complementarity with an RNA splint. Overall, this work serves as a comprehensive framework for assessing the parameters relevant to joining surface-bound ssDNA with externally introduced ssDNA via RNA-splinted ligation.

## Supplementary Material



## Data Availability

Microarray scans and extracted data for all experiments are available upon reasonable request.

## Notes

Supplementary figures and data, tables, oligonucleotide sequences, and additional experimental details for Figure 7 (PDF)

## References

[1] Lackey J. G., Mitra D., Somoza M. M., Cerrina F., Damha M. J. (2009). Acetal
Levulinyl Ester (ALE) Groups for 2′-Hydroxyl Protection of
Ribonucleosides in the Synthesis of Oligoribonucleotides on Glass
and Microarrays. J. Am. Chem. Soc..

[2] Lietard J., Ameur D., Damha M. J., Somoza M. M. (2018). High-Density RNA
Microarrays Synthesized In Situ by Photolithography. Angew. Chem., Int. Ed..

[3] Lietard J., Schaudy E., Hölz K., Ameur D., Somoza M. M. (2019). High-Density
DNA and RNA microarrays - Photolithographic Synthesis, Hybridization
and Preparation of Large Nucleic Acid Libraries. J. Vis. Exp..

[4] Kekić T., Milisavljević N., Troussier J., Tahir A., Debart F., Lietard J. (2024). Accelerated,
high-quality photolithographic synthesis
of RNA microarrays in situ. Sci. Adv..

[5] Lietard J., Damha M. J., Somoza M. M. (2019). Large-Scale Photolithographic Synthesis
of Chimeric DNA/RNA Hairpin Microarrays To Explore Sequence Specificity
Landscapes of RNase HII Cleavage. Biochemistry.

[6] Lietard J., Abou Assi H., Gómez-Pinto I., González C., Somoza M. M., Damha M. J. (2017). Mapping the affinity landscape of
Thrombin-binding aptamers on 2′F-ANA/DNA chimeric G-Quadruplex
microarrays. Nucleic Acids Res..

[7] Hölz K., Pavlic A., Lietard J., Somoza M. M. (2019). Specificity and
Efficiency of the Uracil DNA Glycosylase-Mediated Strand Cleavage
Surveyed on Large Sequence Libraries. Sci. Rep..

[8] Hölz K., Schaudy E., Lietard J., Somoza M. M. (2019). Multi-level patterning
nucleic acid photolithography. Nat. Commun..

[9] Schaudy E., Lietard J., Somoza M. M. (2021). Sequence
Preference and Initiator
Promiscuity for *De Novo* DNA Synthesis by Terminal
Deoxynucleotidyl Transferase. ACS Synth. Biol..

[10] Schaudy E., Somoza M. M., Lietard J. (2020). L-DNA Duplex Formation as a Bioorthogonal
Information Channel in Nucleic Acid-Based Surface Patterning. Chem. – Eur. J..

[11] Ilić I., Schaudy E., Lietard J. (2026). Synthesis of highly complex phosphorothioate-modified
oligonucleotides on microarrays. Sci. Rep..

[12] Agbavwe C., Somoza M. M. (2011). Sequence-Dependent
Fluorescence of Cyanine Dyes on
Microarrays. PLoS One.

[13] Kekić T., Lietard J. (2022). Sequence-dependence
of Cy3 and Cy5 dyes in 3′
terminally-labeled single-stranded DNA. Sci.
Rep..

[14] Lietard J., Ameur D., Somoza M. M. (2022). Sequence-dependent
quenching of fluorescein
fluorescence on single-stranded and double-stranded DNA. RSC Adv..

[15] Schaudy E., Lietard J. (2023). In situ enzymatic template
replication on DNA microarrays. Methods.

[16] Schaudy E., Hölz K., Lietard J., Somoza M. M. (2022). Simple synthesis
of massively parallel RNA microarrays via enzymatic conversion from
DNA microarrays. Nat. Commun..

[17] El-Sagheer A. H., Brown T. (2012). Click Nucleic Acid
Ligation: Applications in Biology and Nanotechnology. Acc. Chem. Res..

[18] Qiu J., El-Sagheer A. H., Brown T. (2013). Solid phase click ligation for the
synthesis of very long oligonucleotides. Chem.
Commun..

[19] Barišić I., Kamleithner V., Schönthaler S., Wiesinger-Mayr H. (2015). Fast and highly
specific DNA-based multiplex detection on a solid support. Appl. Microbiol. Biotechnol..

[20] Wolff N., Barišić I. (2017). Highly Specific
Ligation-dependent
Microarray Detection of Single Nucleotide Polymorphisms. Methods Mol. Biol..

[21] Zhao A., Gray F. C., MacNeill S. A. (2006). ATP^–^ and NAD^+^-dependent DNA ligases share an
essential function in the
halophilic archaeon Haloferax volcanii. Mol.
Microbiol..

[22] Chen H., Du F., Chen G., Streckenbach F., Yasmeen A., Zhao Y., Tang Z. (2014). Template-directed
Chemical Ligation to Obtain 3′-3′
and 5′-5′ Phosphodiester DNA Linkages. Sci. Rep..

[23] Kalinowski M., Haug R., Said H., Piasecka S., Kramer M., Richert C. (2016). Phosphoramidate Ligation of Oligonucleotides in Nanoscale
Structures. ChemBioChem.

[24] Luebke K. J., Dervan P. B. (1991). Nonenzymic sequence-specific
ligation of double-helical
DNA. J. Am. Chem. Soc..

[25] Sokolova N. I., Ashirbekova D. T., Dolinnaya N. G., Shabarova Z. A. (1988). Chemical
reactions within DNA duplexes. Cyanogen bromide as an effective oligodeoxyribonucleotide
coupling agent. FEBS Lett..

[26] Green M. R., Sambrook J. (2019). Ligation and Ligases. Cold Spring
Harb. Protoc..

[27] Pascal J. M. (2008). DNA and
RNA ligases: structural variations and shared mechanisms. Curr. Opin. Struct. Biol..

[28] Pergolizzi G., Wagner G. K., Bowater R. P. (2016). Biochemical
and structural characterization
of DNA ligases from bacteria and archaea. Biosci.
Rep..

[29] Shuman S., Lima C. D. (2004). The polynucleotide
ligase and RNA capping enzyme superfamily
of covalent nucleotidyltransferases. Curr. Opin.
Struct. Biol..

[30] Tomkinson A. E., Vijayakumar S., Pascal J. M., Ellenberger T. (2006). DNA Ligases:
Structure, Reaction Mechanism, and Function. Chem. Rev..

[31] Bullard D. R., Bowater R. P. (2006). Direct comparison of nick-joining activity of the nucleic
acid ligases from bacteriophage T4. Biochem.
J..

[32] Kleppe K., Van de Sande J. H., Khorana H. G. (1970). Polynucleotide Ligase-Catalyzed Joining
of Deoxyribo-oligonucleotides on Ribopolynucleotide Templates and
of Ribo-oligonucleotides on Deoxyribopolynucleotide Templates. Proc. Natl. Acad. Sci. U. S. A..

[33] Lohman G. J. S., Zhang Y., Zhelkovsky A. M., Cantor E. J., Evans T. C. (2014). Efficient
DNA ligation in DNA–RNA hybrid helices by Chlorella virus DNA
ligase. Nucleic Acids Res..

[34] Nilsson M. (2001). RNA-templated
DNA ligation for transcript analysis. Nucleic
Acids Res..

[35] Nilsson M., Barbany G., Antson D.-O., Gertow K., Landegren U. (2000). Enhanced detection
and distinction of RNA by enzymatic probe ligation. Nat. Biotechnol..

[36] Sekiguchi J., Shuman S. (1997). Ligation of RNA-Containing
Duplexes by Vaccinia DNA
Ligase. Biochemistry.

[37] Das A., Santhosh S., Giridhar M., Behr J., Michel T., Schaudy E., Ibáñez-Redín G., Lietard J., Somoza M. M. (2023). Dipodal Silanes Greatly Stabilize
Glass Surface Functionalization for DNA Microarray Synthesis and High-Throughput
Biological Assays. Anal. Chem..

[38] Behr, J. ; Michel, T. ; Giridhar, M. ; Santhosh, S. ; Das, A. ; Sabzalipoor, H. ; Kekić, T. ; Parlar, E. ; Ilić, I. ; Ibáñez-Redín, G. An open-source advanced maskless synthesizer for light-directed chemical synthesis of large nucleic acid libraries and microarrays.ChemRxiv, February 21, 2024. 10.26434/chemrxiv-2024-j4c90.

[39] Michel T., Behr J., Sabzalipoor H., Ibáñez-Redín G., Lietard J., Schletterer T., Funck M., Somoza M. M. (2025). Color-corrected
and high-contrast catadioptric relay for maskless high-resolution
nucleic acid photolithography. Opt. Express.

[40] Santhosh S., Istvánffy S., Sabary O., Yaakobi E., Giridhar M., Behr J., Somoza M. M. (2025). Efficiency of Digital Photolithographic
Synthesis of Large, High-Quality DNA Libraries and Microarrays using
a Guanine O^6^ Dephosphitylation Strategy. Commun. Chem..

[41] Hölz K., Hoi J. K., Schaudy E., Somoza V., Lietard J., Somoza M. M. (2018). High-Efficiency Reverse (5′→3′)
Synthesis of Complex DNA Microarrays. Sci. Rep..

[42] Kretschy N., Holik A.-K., Somoza V., Stengele K.-P., Somoza M. M. (2015). Next-Generation *o*-Nitrobenzyl Photolabile Groups for Light-Directed Chemistry
and Microarray Synthesis. Angew. Chem., Int.
Ed..

[43] Hölz K., Lietard J., Somoza M. M. (2017). High-Power 365 nm UV LED Mercury
Arc Lamp Replacement for Photochemistry and Chemical Photolithography. ACS Sustainable Chem. Eng..

[44] Agbavwe C., Kim C., Hong D., Heinrich K., Wang T., Somoza M. M. (2011). Efficiency,
error and yield in light-directed maskless synthesis of DNA microarrays. J. Nanobiotechnol..

[45] Giacomello S., Lundeberg J. (2018). Preparation of plant tissue to enable Spatial Transcriptomics
profiling using barcoded microarrays. Nat. Protoc..

[46] Fornace M. E., Huang J., Newman C. T., Nanjundiah A., Porubsky N. J., Pierce M. B., Pierce N. A. (2026). NUPACK: Computational
Nucleic Acid Analysis and Design. ACS Synth.
Biol..

[47] Zadeh J. N., Steenberg C. D., Bois J. S., Wolfe B. R., Pierce M. B., Khan A. R., Dirks R. M., Pierce N. A. (2011). NUPACK:
Analysis
and design of nucleic acid systems. J. Comput.
Chem..

[48] Ståhl P. L., Salmén F., Vickovic S., Lundmark A., Navarro J. F., Magnusson J., Giacomello S., Asp M., Westholm J. O., Huss M. (2016). Visualization and analysis of gene expression in tissue
sections by spatial transcriptomics. Science.

[49] Wang G., Moffitt J. R., Zhuang X. (2018). Multiplexed imaging of high-density
libraries of RNAs with MERFISH and expansion microscopy. Sci. Rep..

[50] Vester B., Wengel J. (2004). LNA (Locked Nucleic Acid): High-Affinity
Targeting
of Complementary RNA and DNA. Biochemistry.

[51] Emanuelson C., Bardhan A., Deiters A. (2021). DNA Computing: NOT Logic Gates See
the Light. ACS Synth. Biol..

[52] Hartmann D., Chowdhry R., Smith J. M., Booth M. J. (2023). Orthogonal Light-Activated
DNA for Patterned Biocomputing within Synthetic Cells. J. Am. Chem. Soc..

[53] Dorsey P. J., Rubanov M., Wang W., Schulman R. (2019). Digital Maskless Photolithographic
Patterning of DNA-Functionalized Poly­(ethylene glycol) Diacrylate
Hydrogels with Visible Light Enabling Photodirected Release of Oligonucleotides. ACS Macro Lett..

[54] Huang F., Chen M., Zhou Z., Duan R., Xia F., Willner I. (2021). Spatiotemporal patterning
of photoresponsive DNA-based
hydrogels to tune local cell responses. Nat.
Commun..

[55] Huang F., Xu H., Tan W., Liang H. (2014). Multicolor and Erasable DNA Photolithography. ACS Nano.

[56] Mazzotti G., Hartmann D., Booth M. J. (2023). Precise,
Orthogonal Remote-Control
of Cell-Free Systems Using Photocaged Nucleic Acids. J. Am. Chem. Soc..

[57] Pardatscher G., Schwarz-Schilling M., Daube S. S., Bar-Ziv R. H., Simmel F. C. (2018). Gene Expression
on DNA Biochips Patterned with Strand-Displacement Lithography. Angew. Chem., Int. Ed..

[58] Kohman R. E., Cha S. S., Man H.-Y., Han X. (2016). Light-Triggered Release
of Bioactive Molecules from DNA Nanostructures. Nano Lett..

[59] Everson H. R., Neyra K., Scarton D. V., Chandrasekhar S., Green C. M., Schmidt T.-L., Medintz I. L., Veneziano R., Mathur D. (2024). Purification of DNA Nanoparticles Using Photocleavable
Biotin Tethers. ACS Appl. Mater. Interfaces.

[60] Dobretzberger V., Hebel A., Ponetsmüller M., Ohner F., Umrekar T. R., Beeby M., Simmel F. C., Barišić I. (2026). Bacterial
Expression of Gene-Encoding DNA Origami Structures. Small Struct..

[61] Piranej S., Ogasawara H., Zhang L., Jackson K., Bazrafshan A., Salaita K. (2025). On-Demand Photoactivation of DNA-Based Motor Motion. ACS Nano.

[62] O’Hagan M. P., Duan Z., Huang F., Laps S., Dong J., Xia F., Willner I. (2023). Photocleavable
Ortho-Nitrobenzyl-Protected DNA Architectures
and Their Applications. Chem. Rev..

[63] Zhou Z., Chan H. Y., Lo P. K. (2025). Precision Control of Light-Responsive
Nucleic Acids Modified with Photoremovable Protecting Groups for Functionalization. JACS Au.

[64] Jin J., Vaud S., Zhelkovsky A. M., Posfai J., McReynolds L. A. (2016). Sensitive
and specific miRNA detection method using SplintR Ligase. Nucleic Acids Res..

[65] Franssen-van
Hal N. L. W., van der Putte P., Hellmuth K., Matysiak S., Kretschy N., Somoza M. M. (2013). Optimized Light-Directed Synthesis
of Aptamer Microarrays. Anal. Chem..

[66] Balamurugan S., Obubuafo A., McCarley R. L., Soper S. A., Spivak D. A. (2008). Effect
of Linker Structure on Surface Density of Aptamer Monolayers and Their
Corresponding Protein Binding Efficiency. Anal.
Chem..

[67] Bulyk M. L., Gentalen E., Lockhart D. J., Church G. M. (1999). Quantifying DNA–protein
interactions by double-stranded DNA arrays. Nat. Biotechnol..

[68] Castronovo M., Radovic S., Grunwald C., Casalis L., Morgante M., Scoles G. (2008). Control of Steric Hindrance on Restriction Enzyme Reactions
with Surface-Bound DNA Nanostructures. Nano
Lett..

[69] McCalla S. E., Luryi A. L., Tripathi A. (2009). Steric Effects and Mass-Transfer
Limitations Surrounding Amplification Reactions on Immobilized Long
and Clinically Relevant DNA Templates. Langmuir.

