# Supplementary material for: Quantitative Characterization of RNA-Splinted Ligation as a Multipurpose Post-Synthetic Strategy for High-Density DNA Microarrays Synthesized by Nucleic Acid Photolithography
Source: Anal Chem. 2026 Sep 17;98(38):28299–311. doi: 10.1021/acs.analchem.6c03811 (PMC13629165; doi:10.1021/acs.analchem.6c03811)
Supplement: Supplementary file 1 [file ac6c03811_si_001.pdf]

Supplementary Information

Quantitative Characterization of RNA-Splinted Ligation as a Multipurpose Post-Synthetic Strategy for High-Density DNA Microarrays Synthesized by Nucleic Acid Photolithography

Arya Das[1,2], Mark M. Somoza[2,3,4*]

[1] Technical University of Munich, 85748 Garching, Germany; TUM School of Natural Sciences.

[2] Leibniz Institute for Food Systems Biology at the Technical University of Munich, 85354 Freising, Germany

[3] Chair of Food Chemistry and Molecular Sensory Science, Technical University of Munich, 85354 Freising, Germany

[4] Institute of Inorganic Chemistry, Faculty of Chemistry, University of Vienna, 1090 Vienna, Austria

* mark.somoza@univie.ac.at

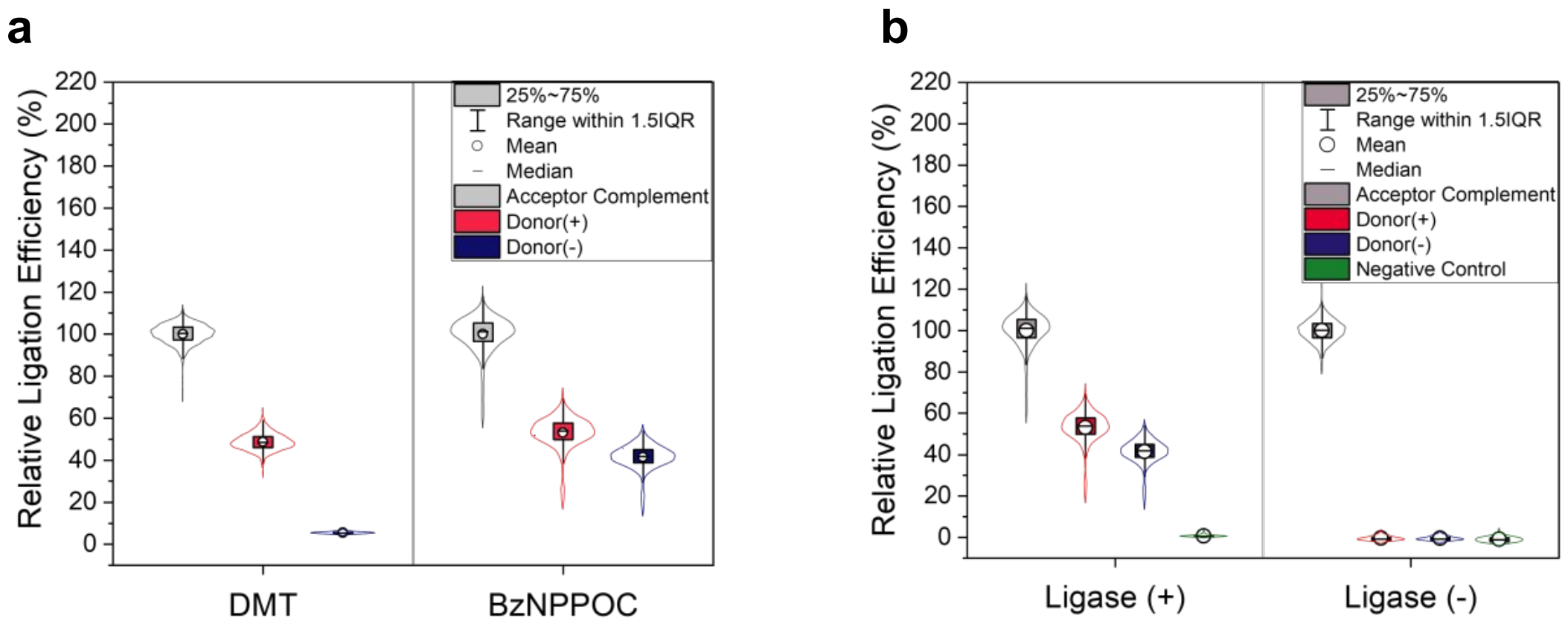

Supplementary Figure S1. a. Unintended ligation on Donor (-) probe in the presence of BzNPPOC-based terminal protection vs absence of ligation in the presence of DMT protection. **b.** Unintended ligation on BzNPPOC-protected probes happens only in the presence of ligase.

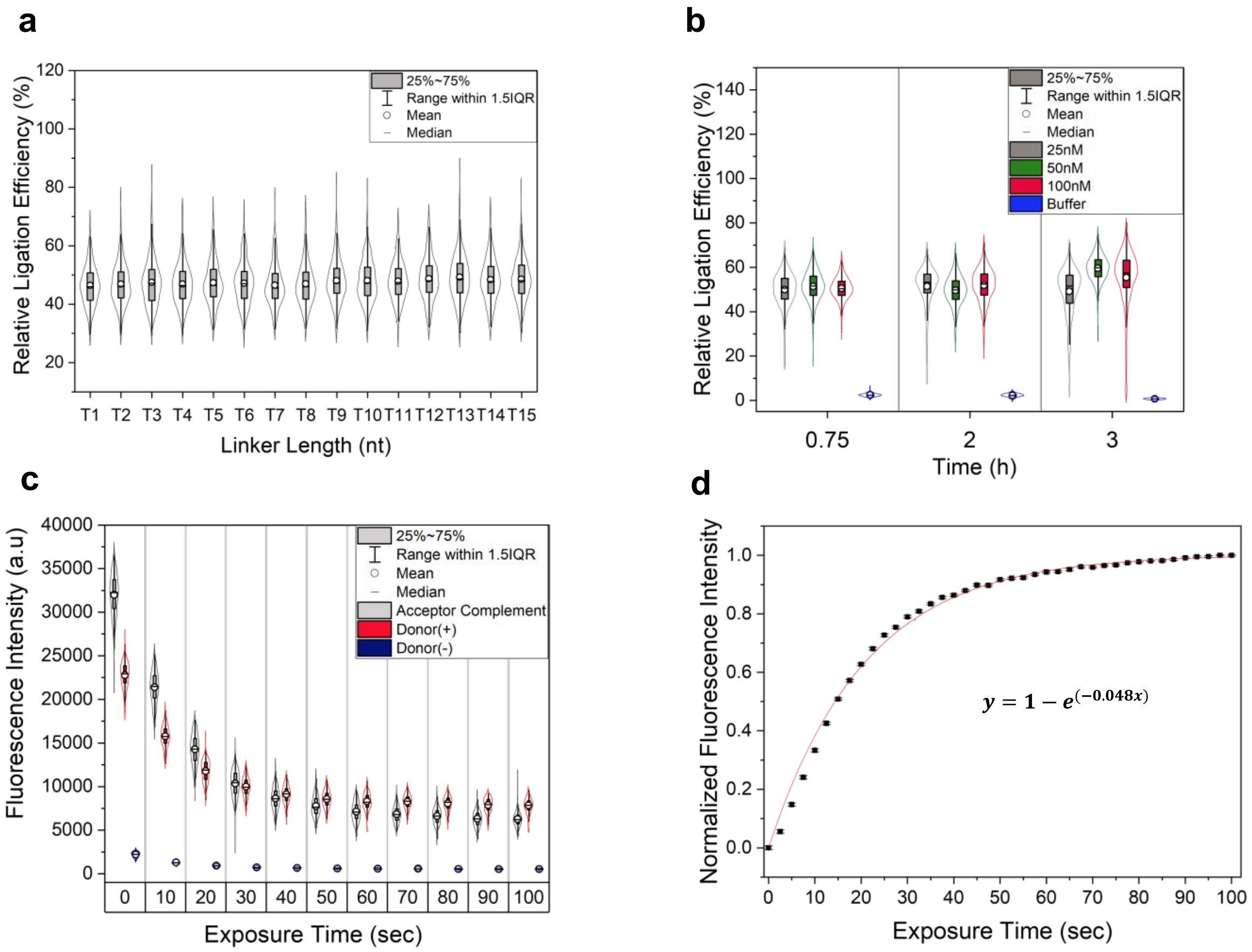

Supplementary Figure S2. Strategies to improve relative ligation efficiency. **a.** Effect of different linker lengths on relative ligation efficiency. **b.** Effect of incubation time and different ligase concentrations. **c.** Raw signal intensity of the sequences after ligation at different UV exposure times. **d.** Single-exponential fit curve showing the variation of signal intensities from the reference sequence at different exposure times. Data normalized to the signal at maximum exposure.

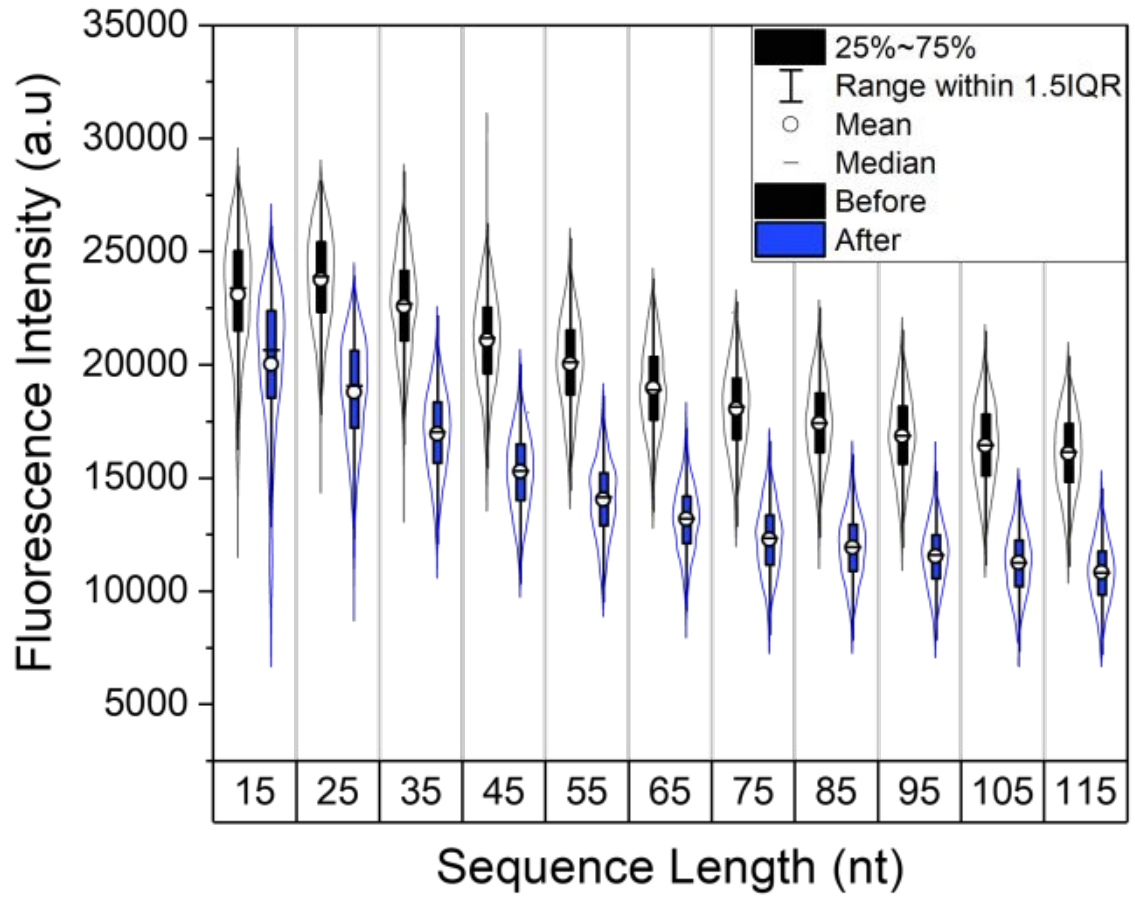

Supplementary Figure S3. Raw signal intensities before and after ligation on sequences of different lengths before and after RNase H treatment.

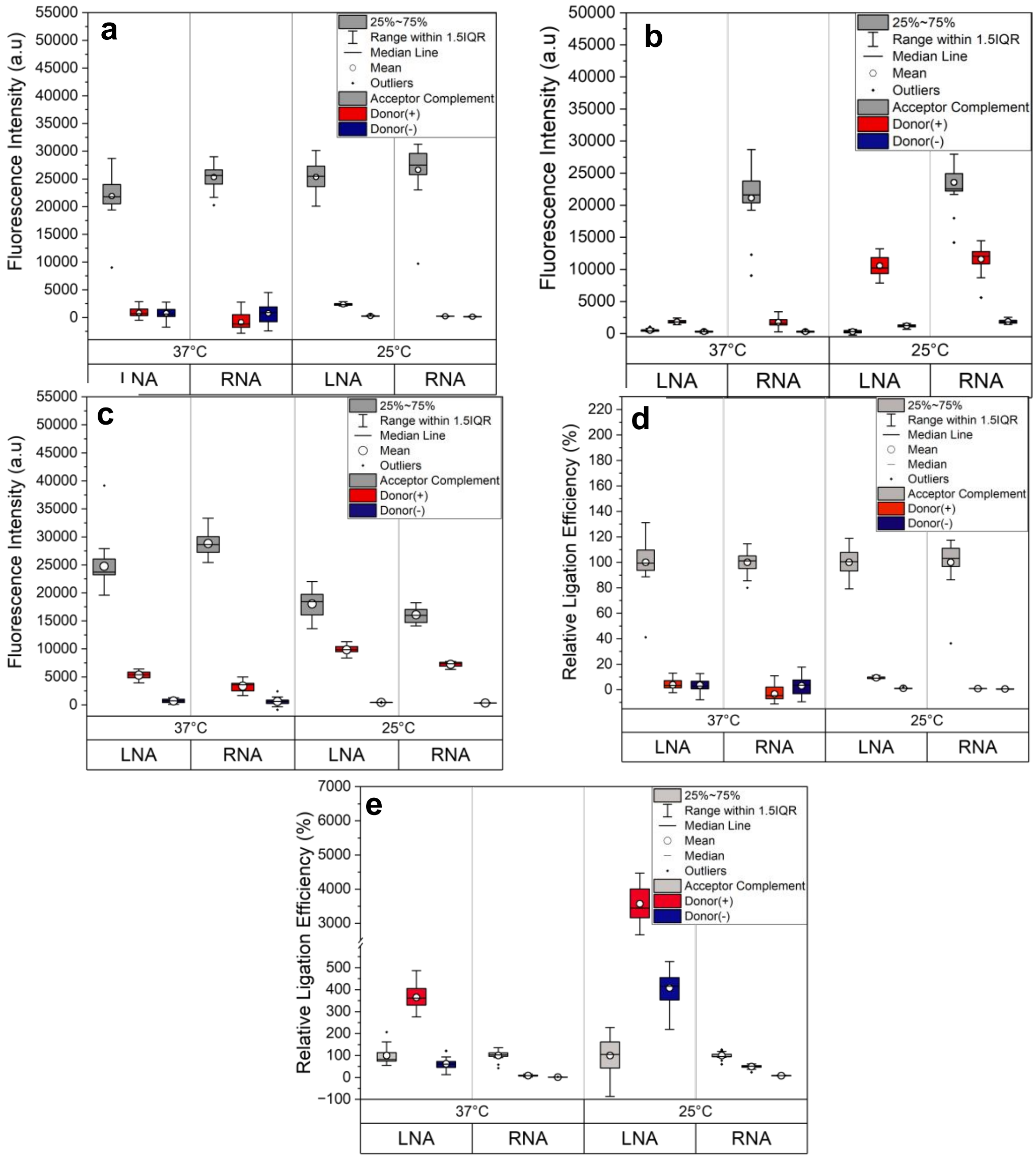

Supplementary Figure S4. Raw fluorescence intensities and relative ligation efficiencies of donor sequences with different GC contents ligated using either an RNA-only splint or a splint containing

a single RNA:LNA substitution at 25 °C and 37 °C. Raw fluorescence intensities for **(a)** Low GC, **(b)** High GC, and **(c)** Moderate GC are shown. Relative ligation efficiencies, calculated by normalizing the ligation signal to the fluorescence signal of the reference probe complementary to the Cy3-labeled acceptor sequence, are shown for **(d)** Low GC and **(e)** High GC. The corresponding data for the Moderate GC sequences are presented in **Figure 4d**. The apparent enhancement observed for the high GC sequences with LNA containing splint results from the unusually low fluorescence signal of the reference probe rather than an increase in the ligation signal (compare **b** and **e**).

a

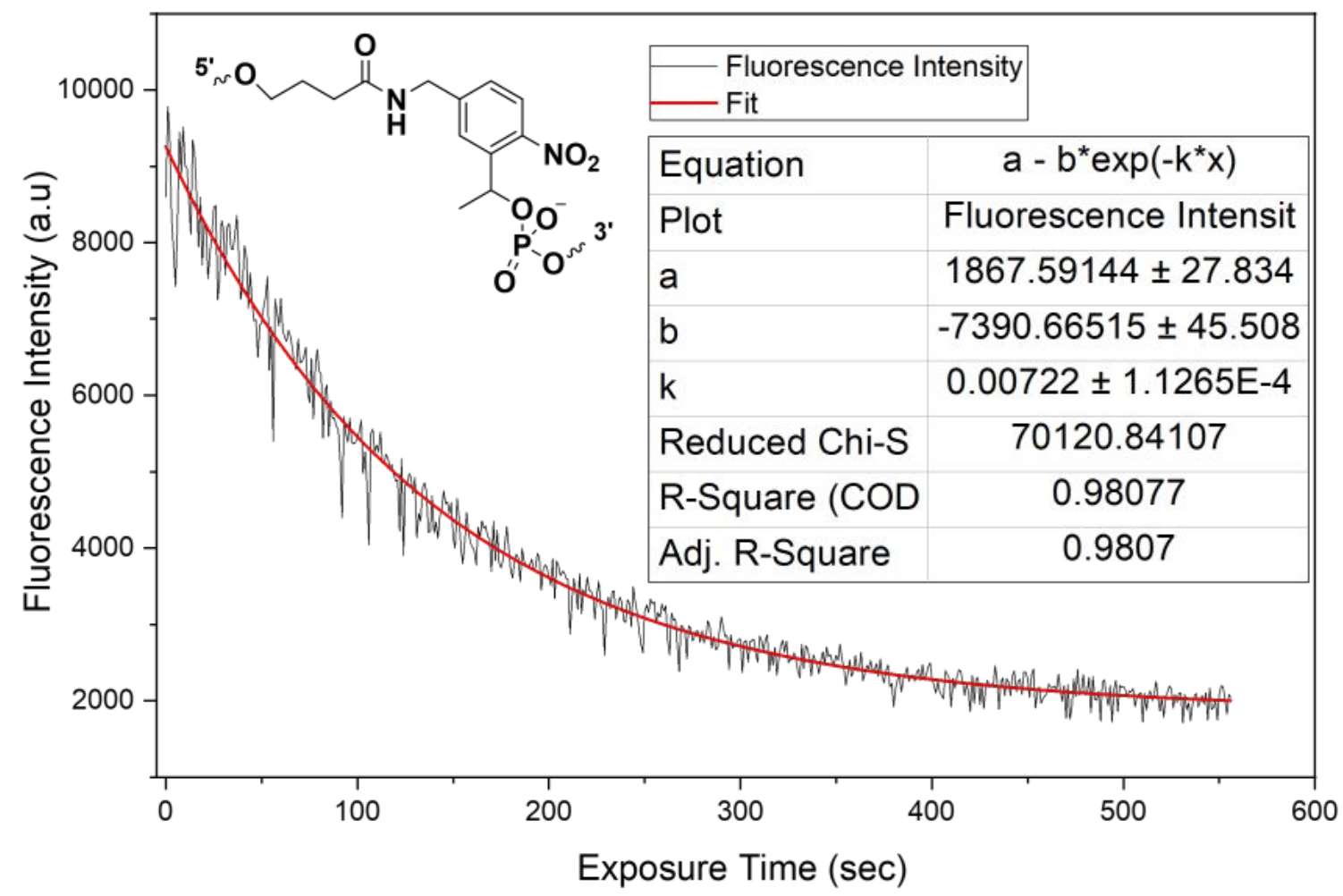

b

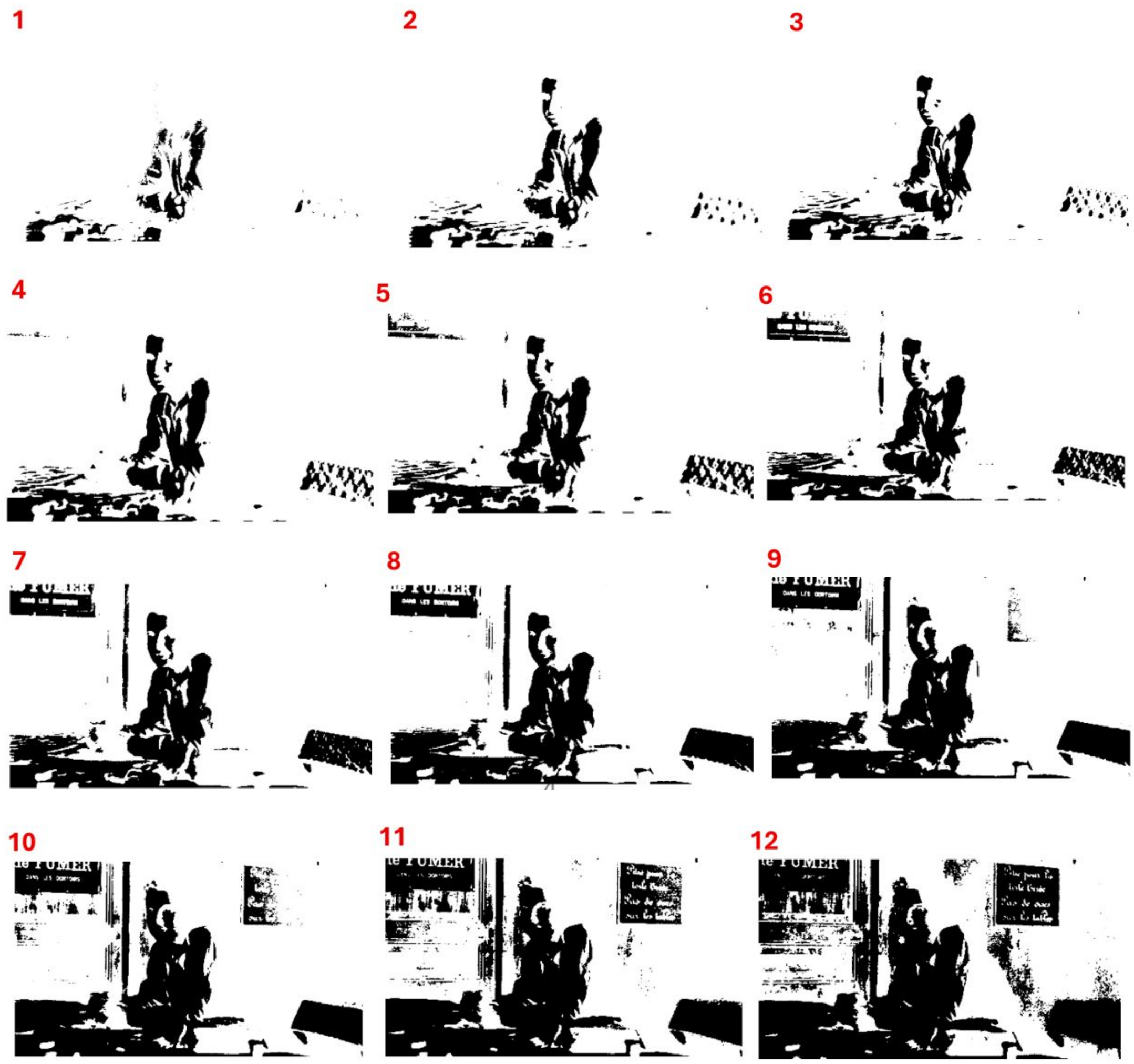

13
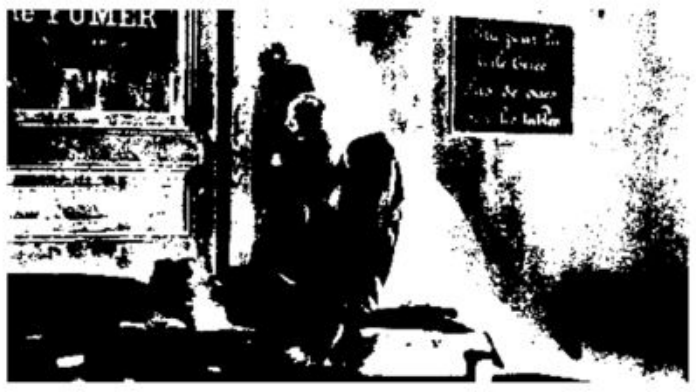
14
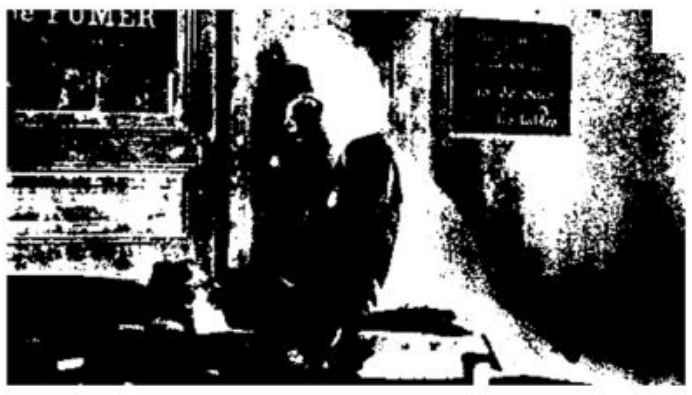
15
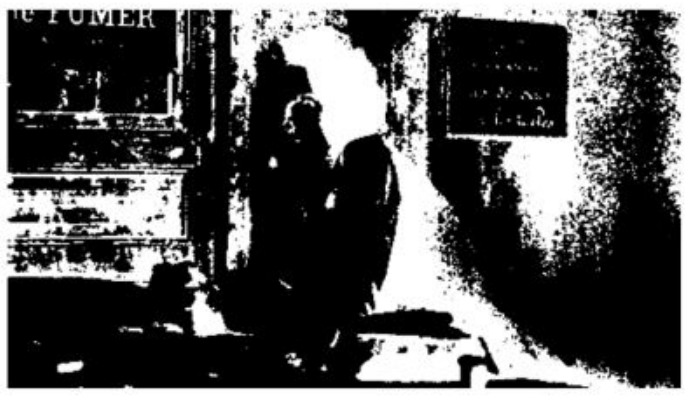
16
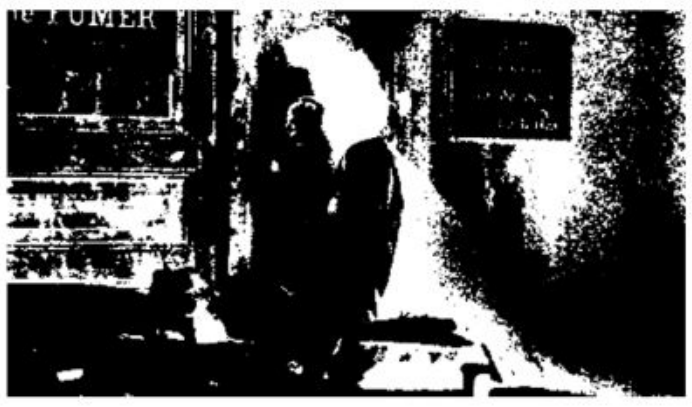
17
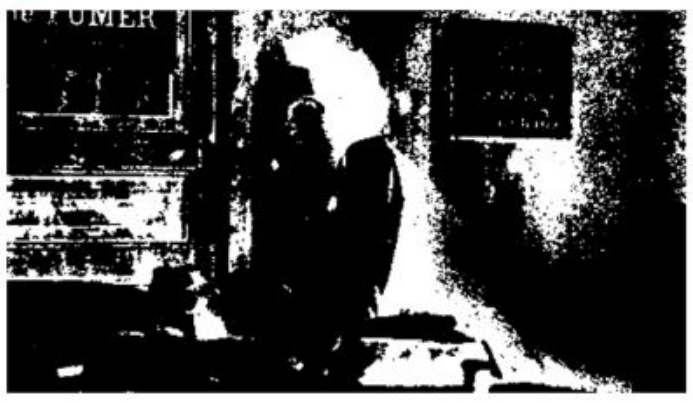
18
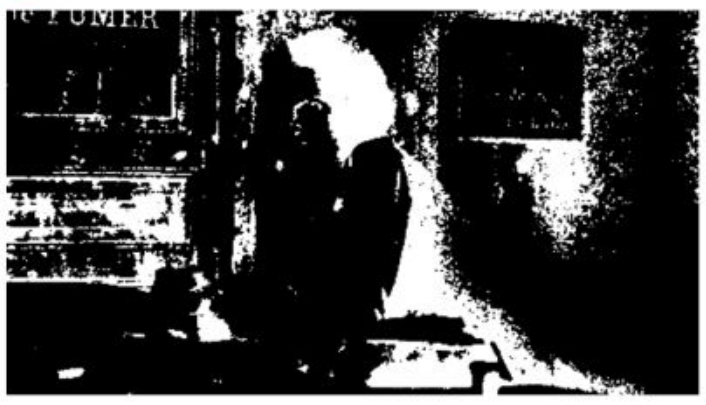
19
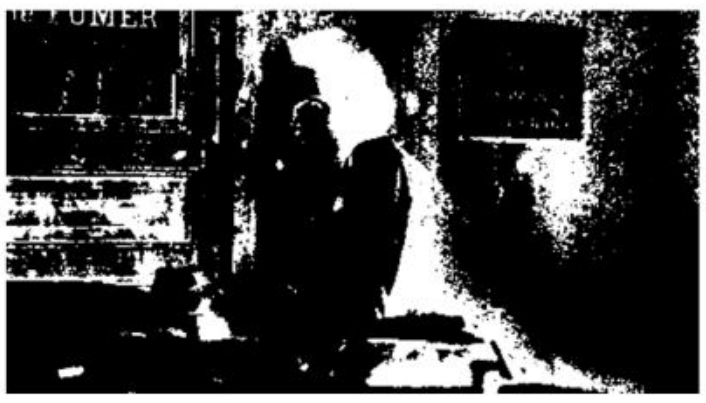
20
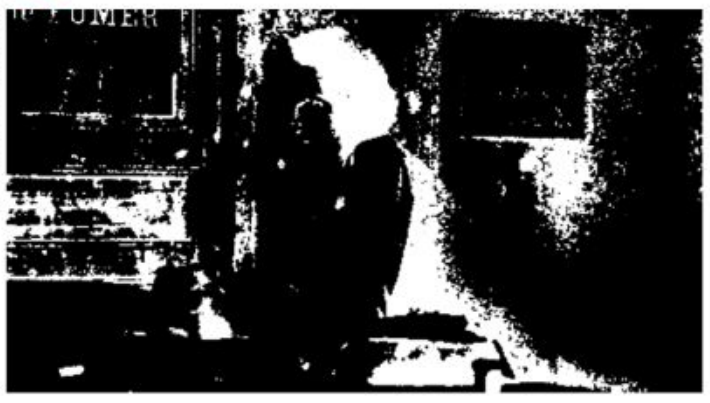
21
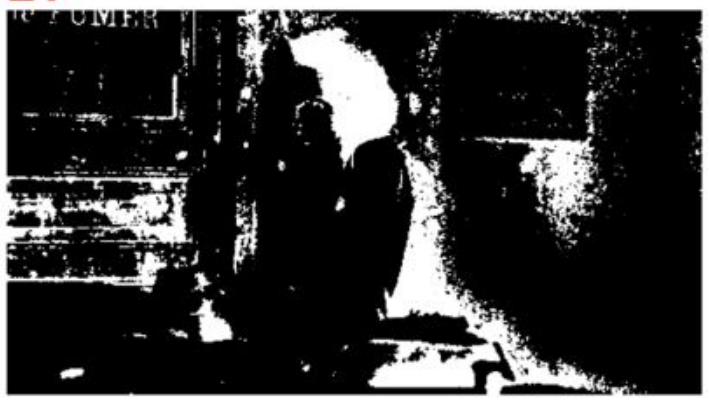
22
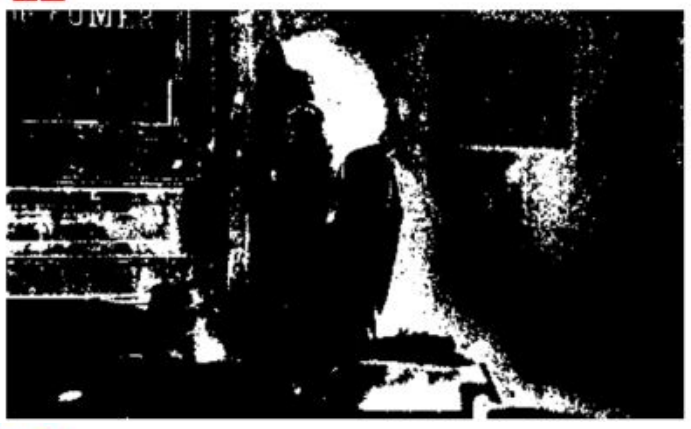
23
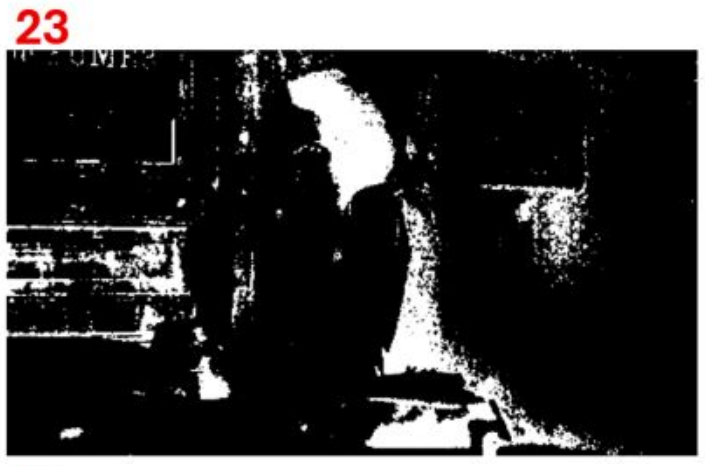
24
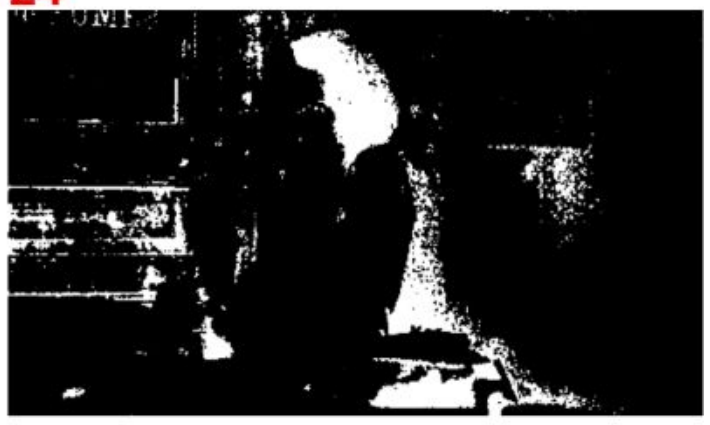
25
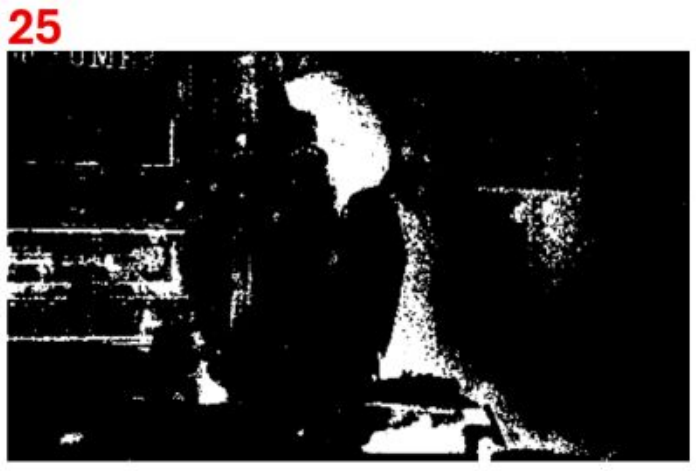
26
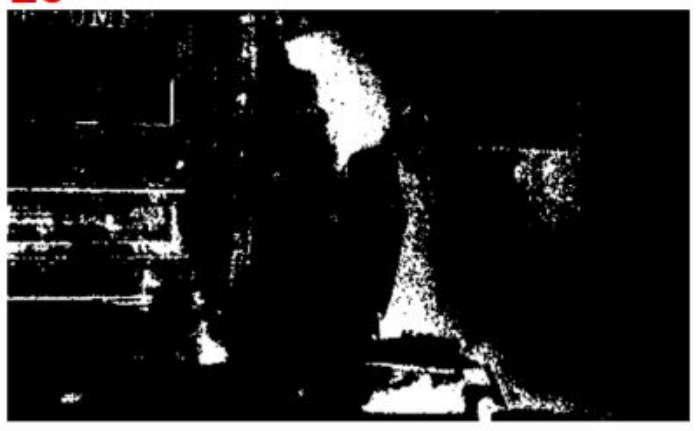
27
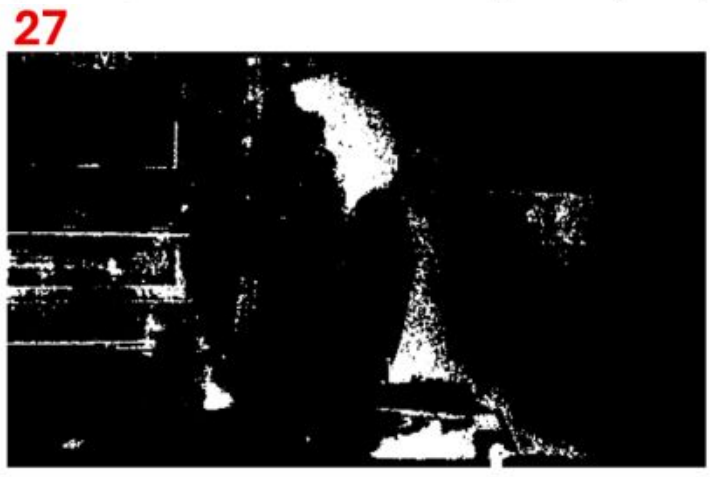
28
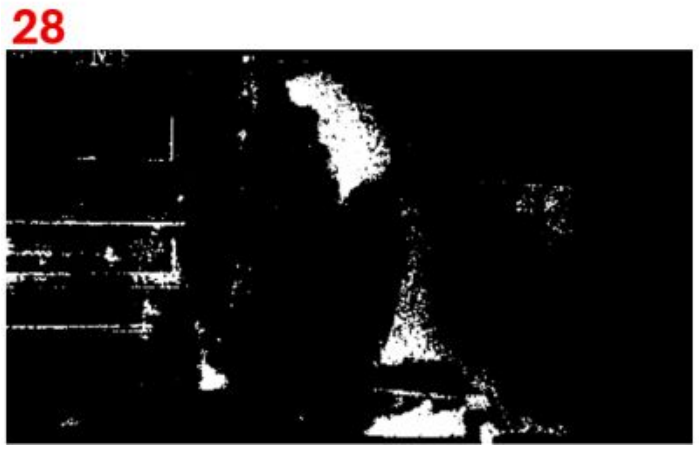
29
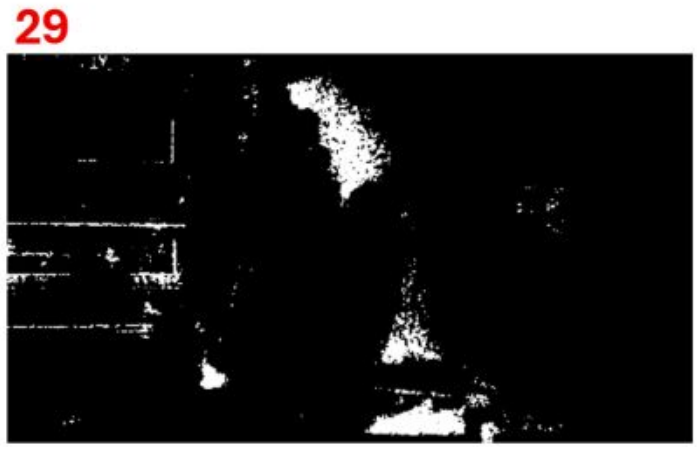
30
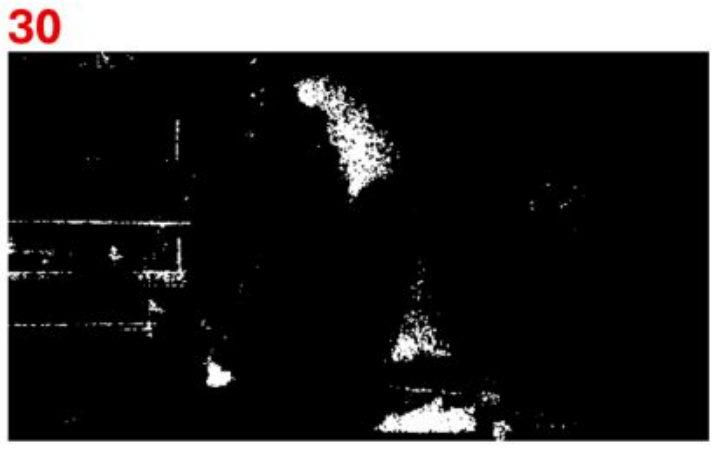

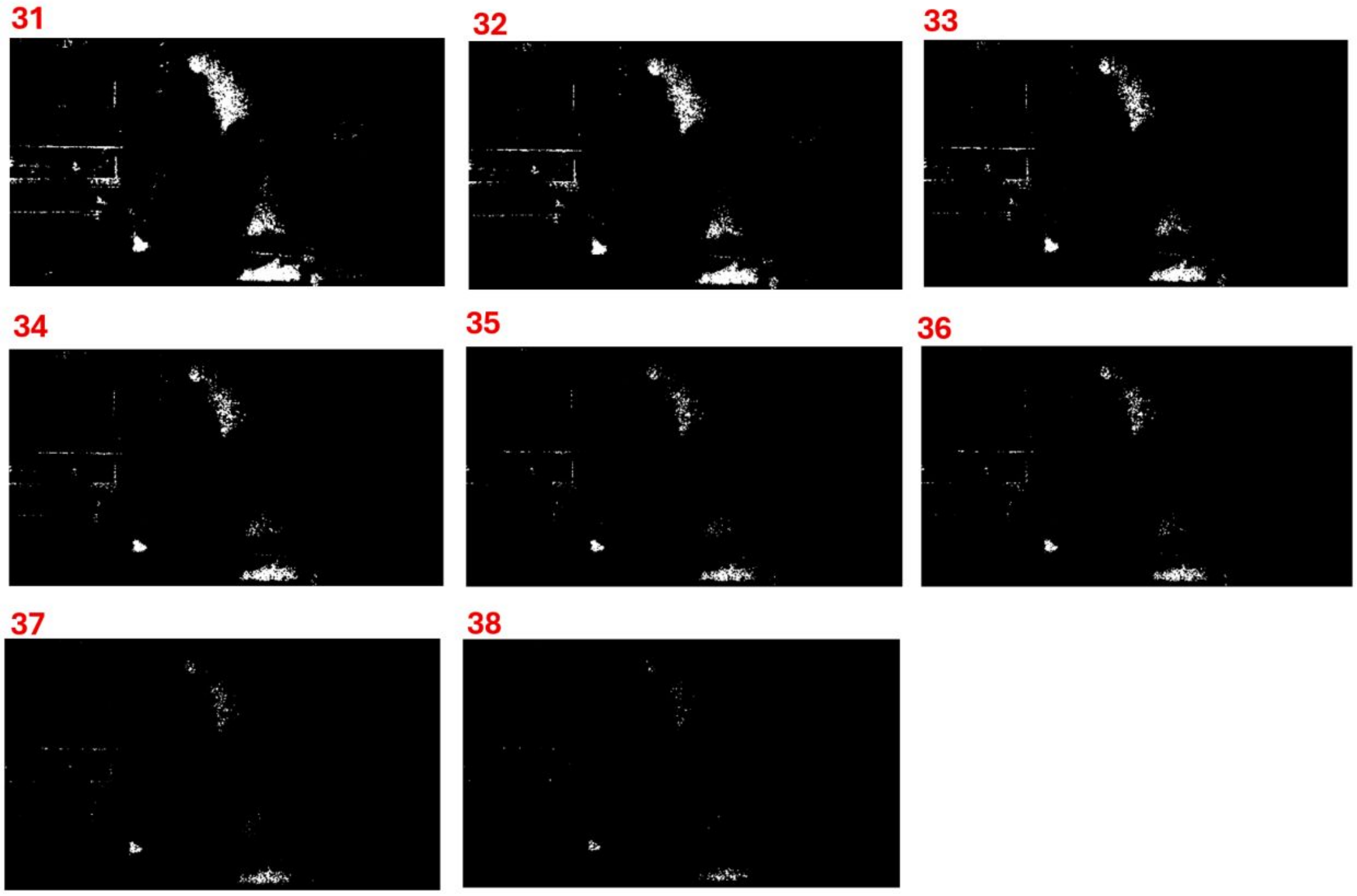

Supplementary Figure S5. **a**. Exposure-dependent loss of fluorescence intensity plotted as a function of exposure time. Data fitted with a first-order kinetics equation. Insert: the chemical structure of the photocleavable spacer. **b**. 38 virtual masks were used to create the exposure patterns to reproduce the original image in Cy3-labeled DNA.

Supplementary Table S1. Sequences used in this study.

Sequences used for Figure 1. PHO indicates phosphate modification.

**Sequence type**	**Sequence name**	**Sequence (5′→3′)**
DNA	Donor(+)	PHO-ATTAACATGAGCGTAGCCAG
DNA	Donor(-)	DMT-ATTAACATGAGCGTAGCCAG
DNA	Acceptor complementary	AAGGGCGACACGGATATGTT
DNA	5'Cy3- acceptor	Cy3-GAAACATATCCGTGTCGCCCTT
DNA	Negative control	PHO-CGGTTGCGTTCATCTATTTC
RNA	RNA splint	GGCUACGCUCAUGUUAAUAAGGGCGACAC GGAUAUGUUUC

Sequences used for Figure 2. PHO indicates phosphate modification, N = A+C+G+T, V = A+C+G

**Sequence type**	**Sequence name**	**Sequence (5′→3′)**
DNA	On array poly (dT) oligo	GTGATCATACCGAATAGGCACTAGTGTGTG ATCAAGAAAACATATCCGTGTCGCCCTTAT TAACATGAGCGTAGCCNNNNNNNNNNTTTT TTTTTTTTTTTTTTVN
DNA	Ligated poly (dT) oligo	GTGATCATACCGAATAGGCACTAGTGTGTG ATCAAGAAAACATATCCGTGTCGCCCTTAT TAACATGAGCGTAGCCNNNNNNNNNNTTTT TTTTTTTTTTTTTTVN
DNA	Acceptor oligo	GTGATCATACCGAATAGGCACTAGTGTGTG ATCAAGAAAACATATCCGTGTCGCCCTT-OH

DNA	Non-ligatable oligo	GTGATCATACCGAATAGGCACTAGTGTGTG ATCAAGAAAACATATCCGTGTCGCCCTT-DMT
DNA	Donor with 3' poly(dT)	PHO-ATTAACATGAGCGTAGCCNNNNNNNNNNTT TTTTTTTTTTTTTTTTVN
DNA	5'Cy3-poly dA	Cy3-GAAAAAAAAAAAAAAAAAA
RNA	RNA splint	GGCUACGCUCAUGUUAAUAAGGGCGACAC GGAUAUGUUUUC

**Sequence type**	**Sequence name**	**Sequence (5′→3′)**
DNA	Donor(+)	PHO-ATTAACATGAGCGTAGCCAG
DNA	Donor(-)	DMT-ATTAACATGAGCGTAGCCAG
DNA	Acceptor complementary	AAGGGCGACACGGATATGTT
DNA	5'Cy3- acceptor	Cy3-GAAACATATCCGTGTCGCCCTT
DNA	Negative control	PHO-CGGTTGCGTTCATCTATTTC
RNA	RNA splint	GGCUACGCUCAUGUUAAUAAGGGCGACAC GGAUAUGUUUUC

Sequences used for Figure 3. PHO indicates phosphate modification.

**Sequence type**	**Sequence name**	**Sequence (5′→3′)**
DNA	115 (Biotin)	Biotin-GAAACATATCCGTGTCGCCCTTATTAACATGAGCGTAGCCCA CTCGAATCACTTGCTCACTGCCCAAAGTCTAACATCGTTCTA CTCGTAGTCATCATCCCACACCACTCTGTTC
DNA	105 (Biotin)	Biotin-GAAACATATCCGTGTCGCCCTTATTAACATGAGCGTAGCCCA CTCGAATCACTTGCTCACTGCCCAAAGTCTAACATCGTTCTA CTCGTAGTCATCATCCCACAC
DNA	95 (Biotin)	Biotin-GAAACATATCCGTGTCGCCCTTATTAACATGAGCGTAGCCCA CTCGAATCACTTGCTCACTGCCCAAAGTCTAACATCGTTCTA CTCGTAGTCAT
DNA	85 (Biotin)	Biotin-GAAACATATCCGTGTCGCCCTTATTAACATGAGCGTAGCCCA CTCGAATCACTTGCTCACTGCCCAAAGTCTAACATCGTTCTA C
DNA	75 (Biotin)	Biotin-GAAACATATCCGTGTCGCCCTTATTAACATGAGCGTAGCCCA CTCGAATCACTTGCTCACTGCCCAAAGTCTAAC
DNA	65 (Biotin)	Biotin-GAAACATATCCGTGTCGCCCTTATTAACATGAGCGTAGCCCA CTCGAATCACTTGCTCACTGCCC
DNA	55 (Biotin)	Biotin-GAAACATATCCGTGTCGCCCTTATTAACATGAGCGTAGCCCA CTCGAATCACTTG
DNA	45 (Biotin)	Biotin-GAAACATATCCGTGTCGCCCTTATTAACATGAGCGTAGCCCA CTC
DNA	35 (Biotin)	Biotin-GAAACATATCCGTGTCGCCCTTATTAACATGAGCG

DNA	25 (Biotin)	Biotin-GAAACATATCCGTGTCGCCCTTATT
DNA	15 (Biotin)	Biotin-GAAACATATCCGTGT
DNA	115 (Splint)	PHO-ATTAACATGAGCGTAGCCCACTCGAATCACTTGCTCACTGCCCAAAGTCTAACATCGTTCTACTCGTAGTCATCATCCCACACCACTCTGTTCGACTAACTACCAACCACTAGAC
DNA	105 (Splint)	PHO-ATTAACATGAGCGTAGCCCACTCGAATCACTTGCTCACTGCCCAAAGTCTAACATCGTTCTACTCGTAGTCATCATCCCACACCACTCTGTTCGACTAACTACCA
DNA	95 (Splint)	PHO-ATTAACATGAGCGTAGCCCACTCGAATCACTTGCTCACTGCCCAAAGTCTAACATCGTTCTACTCGTAGTCATCATCCCACACCACTCTGTTCGA
DNA	85 (Splint)	PHO-ATTAACATGAGCGTAGCCCACTCGAATCACTTGCTCACTGCCCAAAGTCTAACATCGTTCTACTCGTAGTCATCATCCCACACCA
DNA	75 (Splint)	PHO-ATTAACATGAGCGTAGCCCACTCGAATCACTTGCTCACTGCCCAAAGTCTAACATCGTTCTACTCGTAGTCATCA
DNA	65 (Splint)	PHO-ATTAACATGAGCGTAGCCCACTCGAATCACTTGCTCACTGCCCAAAGTCTAACATCGTTCTACTC
DNA	55 (Splint)	PHO-ATTAACATGAGCGTAGCCCACTCGAATCACTTGCTCACTGCCCAAAGTCTAACAT
DNA	45 (Splint)	PHO-ATTAACATGAGCGTAGCCCACTCGAATCACTTGCTCACTGCCCAA
DNA	35 (Splint)	PHO-ATTAACATGAGCGTAGCCCACTCGAATCACTTGCT
DNA	25 (Splint)	PHO-ATTAACATGAGCGTAGCCCACTCGA
DNA	15 (Splint)	PHO-ATTAACATGAGCGTA
DNA	5' Cy3-acceptor	Cy3-GAAACATATCCGTGTCGCCCTT
RNA	RNA splint	CUGGCUACGCUCAUGUUAAUAAGGGCGACACGGAUAUGUU

Sequences used for Figure 4. PHO indicates phosphate modification. Bases in bold case indicate LNA modification.

Sequence type	Sequence name	Sequence (5′→3′)
DNA	Donor_40 nt (+)	PHO-ATTAACATGAGCGTAGCCAG
DNA	Donor_40 nt (-)	DMT-ATTAACATGAGCGTAGCCAG
DNA	Donor_30 nt (+)	PHO-ATTAACATGAGCGTATTTTT
DNA	Donor_30 nt (-)	DMT-ATTAACATGAGCGTATTTTT
DNA	Donor_20 nt (+)	PHO-ATTAACATGATTTTTTTTTT
DNA	Donor_20 nt (-)	DMT-ATTAACATGATTTTTTTTTT
DNA	5'Cy3- acceptor	Cy3-GAAACATATCCGTGTCGCCCTT

DNA	Acceptor complementary	AAGGGCGACACGGATATGTT
RNA	RNA Splint_40 nt	CUGGCUACGCUCAUGUUAAUAAGGGCGACACGGAUAUGUU
RNA	RNA Splint_30 nt	UACGCUCAUGUUAAUAAGGGCGACACGGAU
RNA	RNA Splint_20 nt	UCAUGUUAAUAAGGGCGACA
DNA	5 nt donor_low gc	PHO-ATTAATTTTTTTTTTTTTTTTTTTTTTTTTTTTTTTTTTT
DNA	5 nt donor_moderate gc	PHO-ATTGCTTTTTTTTTTTTTTTTTTTTTTTTTTTTTTTTTTT
DNA	5 nt donor_high gc	PHO-ATCGCTTTTTTTTTTTTTTTTTTTTTTTTTTTTTTTTTTT
DNA	5'Cy3-acceptor_5 nt assay	Cy3-GACCAACGTCATTACCTAGTCTATCTT
DNA	Acceptor complementary_5 nt assay	AAGATAGACTAGGTAATGACGTTGG
RNA	RNA splint_low gc	UUAAUAAGAUAGACUAGGUAAUGACGUUGG
RNA	RNA splint_moderate gc	GCAAUAAGAUAGACUAGGUAAUGACGUUGG
RNA	RNA splint_high gc	GCGAUAAGAUAGACUAGGUAAUGACGUUGG
LNA	LNA splint_low gc	U+**T**AAUAAGAUAGACUAGGUAAUGACGUUGG
LNA	LNA splint_moderate gc	G+**C**AAUAAGAUAGACUAGGUAAUGACGUUGG
LNA	LNA splint_high gc	G+**C**GAUAAGAUAGACUAGGUAAUGACGUUGG

Sequences used for Figure 5. PHO indicates phosphate modification.

**Sequence type**	**Sequence name**	**Sequence (5′→3′)**
DNA	WT_donor	PHO-ATTAACATGAGCGTAGCCAG
DNA	5'Cy3-acceptor	Cy3-GAAACATATCCGTGTCGCCCTT
DNA	WT_acceptor	AACATATCCGTGTCGCCCTT
DNA	3'Cy3-donor	PHO-ATTAACATGAGCGTAGCCAG-Cy3
RNA	RNA splint	CUGGCUACGCUCAUGUUAAUAAGGGCGACACGGAUAUGUU
DNA	Negative control_donor mut	PHO-CGGTTGCGTTCATCTATTTC
DNA	Negative control_acceptor mut	CGGTTGCGTTCATCTATTTC
DNA	Donor mismatch library	Mismatch library of 1771 sequences covering all possible single and double mismatches of 20nt donor sequence.

DNA	Acceptor mismatch library	Mismatch library of 1771 sequences covering all possible single and double mismatches of 20nt acceptor sequence.

Sequences used in Figure 6. N = A+C+G+T and V = A+C+G

Sequence type	Sequence name	Sequence (5′→3′)
DNA	Acceptor oligo	GTGATCATACCGAATAGGCACTAGTGTGTGATCAAGAAAACA TATCCGTGTCGCCCTT-OH
DNA	On array T18VN	GTGATCATACCGAATAGGCACTAGTGTGTGATCAAGAAAACA TATCCGTGTCGCCCTTATTAACATGAGCGTAGCCNNNNNNNN NNTTTTTTTTTTTTTTTTTTVN
DNA	Ligated T18VN	GTGATCATACCGAATAGGCACTAGTGTGTGATCAAGAAAACA TATCCGTGTCGCCCTTATTAACATGAGCGTAGCCNNNNNNNN NNTTTTTTTTTTTTTTTTTTVN
DNA	Control oligo	GTGATCATACCGAATAGGCACTAGTGTGTGATCAAGAAAACA TATCCGTGTCGCCCTT-DMT
DNA	Donor with 3′ T18VN	PHO-ATTAACATGAGCGTAGCCNNNNNNNNNNTTTTTTTTTTTTTTTTTTVN
RNA	RNA splint	GGCUACGCUCAUGUUAAUAAGGG

Sequences used in Figure 7. PC stands for photocleavable spacer. PHO indicates phosphate modification.

Sequence type	Sequence name	Sequence (5′→3′)
Photocleavable spacer modified DNA	5'Cy3-PC-acceptor	Cy3-GATTTTTTTT-PC-AACATATCCGTGTCGCCCTT
DNA	Donor	PHO-ATTAACATGAGCGTAGCCAG
RNA	RNA splint	CUGGCUACGCUCAUGUUAAUAAGGGC GACACGGAUAUGUU

Supplementary Table S2: Conversion of exposure-dependent loss of fluorescence intensity to surface density; Deprotection fraction corresponding to each exposure dose at time, t is calculated as $1-e^{(-kt)}$. The rate constant (k) is calculated as shown in Supplementary Figure S2d. Surface density is calculated as $1-(1-e^{(-kt)})$.

Exposure time (seconds)	Deprotected fraction(%)	Surface density(%)
0	0	100
2.5	11.34120961	88.65879039
5	21.39618886	78.60381114
7.5	30.31081185	69.68918815
10	38.21440875	61.78559125
12.5	45.22164216	54.77835784
15	51.43417054	48.56582946
17.5	56.94212306	43.05787694

20	61.82540714	38.17459286
22.5	66.15486773	33.84513227
25	69.99331513	30.00668487
27.5	73.39643615	26.60356385
30	76.41360209	23.58639791
32.5	79.08858492	20.91141508
35	81.46019234	18.53980766
37.5	83.56283078	16.43716922
40	85.4270046	14.5729954
42.5	87.07975855	12.92024145
45	88.54507022	11.45492978
47.5	89.84419782	10.15580218
50	90.99598863	9.004011372
52.5	92.01715243	7.982847569
55	92.92250391	7.077496093
57.5	93.72517757	6.274822426
60	94.43681834	5.563181662
62.5	95.06775043	4.932249569
65	95.62712719	4.372872807
67.5	96.12306386	3.876936136
70	96.56275532	3.437244682
72.5	96.95258044	3.047419558
75	97.29819468	2.701805318
77.5	97.60461209	2.395387914
80	97.87627805	2.12372195
82.5	98.11713381	1.882866192
85	98.33067361	1.66932639
87.5	98.51999541	1.480004585
90	98.68784584	1.312154163
92.5	98.83665999	1.163340009
95	98.96859682	1.03140318
97.5	99.08557042	0.914429584
100	99.18927779	0.810722208

Supplementary Table S3. Effect of single and double base mismatches on the **(a**, **b)** donor and **(c**, **d)** acceptor side of ligation junction. Signal intensities were normalized to the perfect match sequence in both cases and ranked accordingly. **a** and **c.** Represent the sequences that were highly ligated (10%), **b** and **d.** Represent the sequences that were least ligated (10%).

a

**Sequence**	**Normalized fluorescence intensity±SEM**
ATTAACATGAagGTAGCCAG	1.063 ± 0.013
ATTAACATGAGCGTAaCCgG	1.058 ± 0.012
ATTAACATGAaaGTAGCCAG	1.053 ± 0.013
ATTAACATGAcCGTAGCCAG	1.053 ± 0.016

ATTAACATGAGgGTAGCCAG	1.050 ± 0.011
ATTAACATGAGCGTAGgaAG	1.045 ± 0.019
ATTAACATGAGCGTAGaaAG	1.044 ± 0.015
ATTAACATGAcgGTAGCCAG	1.042 ± 0.012
ATTAACATGAaCGTAGCCAG	1.037 ± 0.015
ATTAACATGgaCGTAGCCAG	1.037 ± 0.015
ATTAACATGAaCGTAGCCAa	1.034 ± 0.015
ATTAACATGAGCGTAcCCcG	1.033 ± 0.023
ATTAACATGAcCGTAGCCAa	1.031 ± 0.010
ATTAACATGAGCGTAGgtAG	1.030 ± 0.017
ATTAACATGAGCGTAaCCcG	1.029 ± 0.010
ATTAACATGAGgtTAGCCAG	1.029 ± 0.016
ATTAACATGAGgGaAGCCAG	1.027 ± 0.018
ATTAACATGAcCGTAGCCAt	1.024 ± 0.012
ATTAACATGAaCGTAGCCAt	1.024 ± 0.016
ATTAACATGgGtGTAGCCAG	1.023 ± 0.020
ATTAACATGtcCGTAGCCAG	1.023 ± 0.017
ATTAACATGAGCGTAcCCgG	1.022 ± 0.018
ATTAACATGAcCGTAGCCAc	1.020 ± 0.018
ATTAACATGAaCGTAGCCcG	1.020 ± 0.018
ATTAACATGttCGTAGCCAG	1.020 ± 0.015
ATTAACATGAGCGgAaCCAG	1.018 ± 0.015
ATTAACATGAcCGTAGCCcG	1.016 ± 0.017
ATTAACATaAcCGTAGCCAG	1.016 ± 0.016
ATTAACATaAGCGTAGCCAG	1.016 ± 0.011
ATTAACATGAGCGTAaCCAG	1.016 ± 0.016
ATTAACATGAGCGTAGtaAG	1.013 ± 0.013
ATTAACATGAcaGTAGCCAG	1.013 ± 0.019
ATTAACATGAGCGTAGCCct	1.012 ± 0.008
ATTAACATGAtaGTAGCCAG	1.012 ± 0.014
ATTAACATGAGCGTAaCCAt	1.012 ± 0.012
ATTAACATGccCGTAGCCAG	1.010 ± 0.017
ATTAACATGAGCGTAcCaAG	1.010 ± 0.012
ATTAACATGAaCGgAGCCAG	1.010 ± 0.012
ATTAACATGgGgGTAGCCAG	1.010 ± 0.015
ATTAACATGAaCGTAGCCAc	1.008 ± 0.018
ATTAACATGAGCGTAaCCAc	1.007 ± 0.013
ATTAACATGgtCGTAGCCAG	1.007 ± 0.010
ATTAACATGAGCGgAcCCAG	1.007 ± 0.016
ATTAACATGAGCcTAGCCcG	1.007 ± 0.014
ATTAACATGAGCGTAaCCAa	1.007 ± 0.017
ATTAACATGAGCGTAGaCAc	1.006 ± 0.012
ATTAACATGAGCGaAaCCAG	1.006 ± 0.017
ATTAACATGgcCGTAGCCAG	1.006 ± 0.014
ATTAACATGgGaGTAGCCAG	1.006 ± 0.011

ATTAACATaAGCGTAGCCAc	1.005 ± 0.013
ATTAACATGAtCGTAGCCAG	1.004 ± 0.022
ATTAACATGAGgGgAGCCAG	1.004 ± 0.018
ATTAACATGAGgGTAGaCAG	1.002 ± 0.011
ATTAACATGAGgGTAGCCAa	1.002 ± 0.011
ATTAACATGAaCGTAGCCgG	1.002 ± 0.013
ATTAACATtAaCGTAGCCAG	1.002 ± 0.014
ATTAACATGAGCGTAGCaAG	1.002 ± 0.013
ATTAACATGAaCGTAGCCtG	1.001 ± 0.014
ATTAACATGAGCGTtaCCAG	1.001 ± 0.014
ATTAACATGtGgGTAGCCAG	1.001 ± 0.018
ATTAACATGAGCcTAaCCAG	1.000 ± 0.012
ATTAACATGAGCGTAGCCAG	1.000 ± 0.025
ATTAACAgaAGCGTAGCCAG	1.000 ± 0.015
ATTAACATGAGCGTAGaCcG	0.999 ± 0.016
ATTAACATGAGCGaAcCCAG	0.999 ± 0.016
ATTAACATGAGgGTAaCCAG	0.999 ± 0.012
ATTAACATGAGatTAGCCAG	0.998 ± 0.017
ATTAACATGAGCGTAGaCgG	0.998 ± 0.012
ATTAACATGtaCGTAGCCAG	0.998 ± 0.013
ATTAACATGAGCGTtGCCcG	0.998 ± 0.016
ATTAACATGgGCGTAaCCAG	0.998 ± 0.011
ATTAACATGAGCGTAGttAG	0.997 ± 0.011
ATTAACATaAGgGTAGCCAG	0.997 ± 0.017
ATTAACATaAaCGTAGCCAG	0.997 ± 0.014
ATTAACATGAcCGaAGCCAG	0.996 ± 0.012
ATTAACATGAGaGaAGCCAG	0.996 ± 0.007
ATTAACATGAcCGTAGCCtG	0.996 ± 0.015
ATTAACATGAGCGTAGCCtt	0.995 ± 0.014
ATTAACATGAGaGTAGCCAG	0.995 ± 0.015
ATTAACATGAGCGTAcCCAa	0.995 ± 0.015
ATTAACATGAGCGTAaaCAG	0.995 ± 0.017
ATTAACATGAGCtTAGCCAG	0.994 ± 0.015
ATTAACATGAGCGTAGaCtG	0.994 ± 0.026
ATTAACATaAGaGTAGCCAG	0.994 ± 0.016
ATTAACATGgGCGTAGtCAG	0.993 ± 0.014
ATTAACATaAGCGTAGCCAa	0.993 ± 0.014
ATTAACATGAGCGTAcCgAG	0.993 ± 0.010
ATTAACATGAGCcaAGCCAG	0.992 ± 0.015
ATTAACATacGCGTAGCCAG	0.992 ± 0.015
ATTAACATGAGCaaAGCCAG	0.992 ± 0.012
ATTAACATGAGCGTAcCCAc	0.992 ± 0.013
ATTAACATGAGCtTAGCCcG	0.992 ± 0.013
ATTAACATaAGtGTAGCCAG	0.991 ± 0.014
ATTAACATGAGCGTAGCaAa	0.991 ± 0.017

ATTAACATGtGaGTAGCCAG	0.991 ± 0.015
ATTAACATGAGCGTAGCCcc	0.990 ± 0.015
ATTAACATGAGCGTAGtCcG	0.990 ± 0.014
ATTAACATGAGCGTAaCaAG	0.989 ± 0.012
ATTAACAcGAGCGTAGCCAG	0.989 ± 0.016
ATTAACATGAGgaTAGCCAG	0.989 ± 0.014
ATTAACATGAGgGTAGgCAG	0.989 ± 0.021
ATTAACATGAGCGaAGCCcG	0.988 ± 0.011
ATTAACATGcaCGTAGCCAG	0.988 ± 0.012
ATTAACATGAGCGagGCCAG	0.987 ± 0.013
ATTAACATGAGCGTAGgCcG	0.987 ± 0.011
ATTAACATGAGgGTAGCCAc	0.987 ± 0.015
ATTAACAcGAGCGTAGCCAa	0.986 ± 0.012
ATTAACATtAGCGTAGCCAG	0.986 ± 0.013
ATTAACATGAGCGTAGaCAa	0.986 ± 0.015
ATTAACATGAGCGTAGCCAc	0.985 ± 0.014
ATTAACATGAGacTAGCCAG	0.985 ± 0.016
ATTAACATGAGCGTgaCCAG	0.985 ± 0.012
ATTAACATGAGCGTAaCtAG	0.985 ± 0.020
ATTAACATGAGCGTAaCgAG	0.984 ± 0.017
ATTAACATGAGCGTAcCtAG	0.982 ± 0.012
ATTAACATGAGCGgAGCCAc	0.982 ± 0.011
ATTAACATGAGCGaAGCCAG	0.982 ± 0.011
ATTAACATGAGCGTAcCCAt	0.981 ± 0.016
ATTAACATtAGCGTAGCCAa	0.981 ± 0.013
ATTAACATGAGgGTAGCCtG	0.980 ± 0.012
ATTAACATGAttGTAGCCAG	0.980 ± 0.015
ATTAACATGAcCGgAGCCAG	0.980 ± 0.012
ATTAACATGAGaGgAGCCAG	0.980 ± 0.015
ATTAACATGAGgGTAGtCAG	0.979 ± 0.012
ATTAACATGAGCGTAGCtAa	0.979 ± 0.013
ATTAACATGgGCGTAGCCgG	0.979 ± 0.011
ATTAACATtAcCGTAGCCAG	0.979 ± 0.011
ATTAACAgGAGCGTAGaCAG	0.979 ± 0.010
ATTAACATGAGaGTAGCCAt	0.978 ± 0.011
ATTAACATGAGtGTAGCCAc	0.978 ± 0.018
ATTAACATGAGCtTAGCCAc	0.978 ± 0.009
ATTAACATGAGCGTAcCCAG	0.978 ± 0.016
ATTAACAgGAGaGTAGCCAG	0.978 ± 0.016
ATTAACATcAaCGTAGCCAG	0.978 ± 0.014
ATTAACATGcGgGTAGCCAG	0.978 ± 0.014
ATTAACATtAtCGTAGCCAG	0.978 ± 0.013
ATTAACATGAGCGggGCCAG	0.977 ± 0.010
ATTAACAaGAGCGTAGCCAa	0.977 ± 0.011
ATTAACATGAGtaTAGCCAG	0.977 ± 0.015

ATTAACATGActGTAGCCAG	0.977 ± 0.018
ATTAACAaGAGCGTAGCCtG	0.977 ± 0.017
ATTAACATGAatGTAGCCAG	0.977 ± 0.013
ATTAACAcGAGCGTAGCCAt	0.977 ± 0.012
ATTAACATGAGCcTAGCCAa	0.976 ± 0.012
ATTAACATGgGCGTAGCCAa	0.976 ± 0.013
ATTAACATGAGtGaAGCCAG	0.976 ± 0.008
ATTAACATGAGCGTAGggAG	0.976 ± 0.012
ATTAACATGAGCGTAGCCcG	0.976 ± 0.014
ATTAACATGAGCGTAGtCgG	0.976 ± 0.016
ATTAACATGAGCGTccCCAG	0.975 ± 0.012
ATTAACATGctCGTAGCCAG	0.975 ± 0.014
ATTAACATcAcCGTAGCCAG	0.975 ± 0.014
ATTAACATGAGaGTAGCCAa	0.975 ± 0.012
ATTAACATGgGCGgAGCCAG	0.975 ± 0.013
ATTAACATGAGaaTAGCCAG	0.975 ± 0.020
ATTAACATGAGCGTAGCCga	0.975 ± 0.015
ATTAACATGAGCGTAGCCgt	0.975 ± 0.018
ATTAACATGAGCccAGCCAG	0.974 ± 0.012
ATTAACATGAGttTAGCCAG	0.974 ± 0.017
ATTAACATGAGgGTAGCCAt	0.974 ± 0.017
ATTAACATGgGCGTAGaCAG	0.974 ± 0.012
ATTAACATGgGCcTAGCCAG	0.974 ± 0.011
ATTAACATGAGgGTAGCaAG	0.974 ± 0.011
ATTAACAaGAGCGTAGCCAG	0.974 ± 0.013
ATTAACATGgGCGTAGgCAG	0.974 ± 0.015
ATTAACATGAGCGaAGCCAa	0.973 ± 0.014
ATTAACAgGAGCGTAGCCAa	0.973 ± 0.015
ATTAACATGAGCGTgGCCAa	0.973 ± 0.018
ATTAACATGAGtGTAGCCAG	0.973 ± 0.015
ATTAACATccGCGTAGCCAG	0.973 ± 0.012
ATTAACATaAGCGTAGCCtG	0.973 ± 0.012
ATTAACATGAGtGTAaCCAG	0.973 ± 0.012
ATTAACATGAGaGTAGCCAc	0.973 ± 0.013
ATTAACATGAGCGTAGatAG	0.973 ± 0.014
ATTAACATagGCGTAGCCAG	0.973 ± 0.018
ATTAACATGAtCGTAGCCAa	0.972 ± 0.013
ATTAACAaGAGCGTAGCCAc	0.972 ± 0.011

b

**Sequence**	**Normalized fluorescence intensity±SEM**
ccTAACATGAGCGTAGCCAG	0.002 ± 0.002
cTaAACATGAGCGTAGCCAG	0.003 ± 0.001
cgTAACATGAGCGTAGCCAG	0.003 ± 0.002

AcgAACATGAGCGTAGCCAG	0.003 ± 0.001
cTcAACATGAGCGTAGCCAG	0.003 ± 0.001
ggTAACATGAGCGTAGCCAG	0.004 ± 0.001
AgcAACATGAGCGTAGCCAG	0.004 ± 0.001
cTgAACATGAGCGTAGCCAG	0.004 ± 0.001
AacAACATGAGCGTAGCCAG	0.004 ± 0.001
cTTcACATGAGCGTAGCCAG	0.004 ± 0.001
gTgAACATGAGCGTAGCCAG	0.005 ± 0.001
gcTAACATGAGCGTAGCCAG	0.006 ± 0.002
AccAACATGAGCGTAGCCAG	0.006 ± 0.003
tcTAACATGAGCGTAGCCAG	0.007 ± 0.001
AagAACATGAGCGTAGCCAG	0.007 ± 0.001
AggAACATGAGCGTAGCCAG	0.007 ± 0.001
AcTAcCATGAGCGTAGCCAG	0.008 ± 0.001
AaaAACATGAGCGTAGCCAG	0.008 ± 0.001
tgTAACATGAGCGTAGCCAG	0.008 ± 0.001
cTTAcCATGAGCGTAGCCAG	0.008 ± 0.002
caTAACATGAGCGTAGCCAG	0.008 ± 0.001
gTcAACATGAGCGTAGCCAG	0.009 ± 0.001
cTTAgCATGAGCGTAGCCAG	0.010 ± 0.001
AcaAACATGAGCGTAGCCAG	0.010 ± 0.001
AcTcACATGAGCGTAGCCAG	0.011 ± 0.001
gTaAACATGAGCGTAGCCAG	0.011 ± 0.003
AcTAtCATGAGCGTAGCCAG	0.011 ± 0.001
AcTAgCATGAGCGTAGCCAG	0.012 ± 0.001
cTTAtCATGAGCGTAGCCAG	0.013 ± 0.001
cTTtACATGAGCGTAGCCAG	0.013 ± 0.002
AgTcACATGAGCGTAGCCAG	0.013 ± 0.002
AgTAcCATGAGCGTAGCCAG	0.014 ± 0.002
AgTAtCATGAGCGTAGCCAG	0.014 ± 0.001
gaTAACATGAGCGTAGCCAG	0.014 ± 0.001
AgaAACATGAGCGTAGCCAG	0.015 ± 0.002
AcTgACATGAGCGTAGCCAG	0.015 ± 0.001
cTTgACATGAGCGTAGCCAG	0.015 ± 0.002
AgTgACATGAGCGTAGCCAG	0.016 ± 0.001
AcTAACcTGAGCGTAGCCAG	0.016 ± 0.001
cTTAACcTGAGCGTAGCCAG	0.016 ± 0.001
AgTAgCATGAGCGTAGCCAG	0.018 ± 0.002
AgTAACcTGAGCGTAGCCAG	0.019 ± 0.002
AaTcACATGAGCGTAGCCAG	0.019 ± 0.002
gTTAACcTGAGCGTAGCCAG	0.020 ± 0.001
AgTAAgATGAGCGTAGCCAG	0.020 ± 0.001
AgTAAaATGAGCGTAGCCAG	0.020 ± 0.002
tTcAACATGAGCGTAGCCAG	0.021 ± 0.001
gTTAcCATGAGCGTAGCCAG	0.021 ± 0.002

AcTAAgATGAGCGTAGCCAG	0.022 ± 0.001
cTTAACtTGAGCGTAGCCAG	0.023 ± 0.001
gTTAgCATGAGCGTAGCCAG	0.023 ± 0.001
AaTgACATGAGCGTAGCCAG	0.023 ± 0.001
AcTAAaATGAGCGTAGCCAG	0.024 ± 0.002
gTTcACATGAGCGTAGCCAG	0.026 ± 0.002
gTTAtCATGAGCGTAGCCAG	0.026 ± 0.002
tTTAACcTGAGCGTAGCCAG	0.027 ± 0.001
AcTAACtTGAGCGTAGCCAG	0.027 ± 0.002
AcTtACATGAGCGTAGCCAG	0.028 ± 0.009
AgTAACtTGAGCGTAGCCAG	0.029 ± 0.002
AaTAcCATGAGCGTAGCCAG	0.029 ± 0.007
gTTtACATGAGCGTAGCCAG	0.030 ± 0.002
tTaAACATGAGCGTAGCCAG	0.031 ± 0.002
AaTAtCATGAGCGTAGCCAG	0.031 ± 0.002
ATacACATGAGCGTAGCCAG	0.033 ± 0.001
tTTAcCATGAGCGTAGCCAG	0.034 ± 0.002
gTTAACtTGAGCGTAGCCAG	0.035 ± 0.002
tTTAgCATGAGCGTAGCCAG	0.035 ± 0.001
taTAACATGAGCGTAGCCAG	0.037 ± 0.002
tTTAACtTGAGCGTAGCCAG	0.037 ± 0.001
AgTtACATGAGCGTAGCCAG	0.037 ± 0.002
AaTAgCATGAGCGTAGCCAG	0.038 ± 0.002
tTTcACATGAGCGTAGCCAG	0.039 ± 0.001
cTTAAaATGAGCGTAGCCAG	0.039 ± 0.005
tTgAACATGAGCGTAGCCAG	0.041 ± 0.005
tTTgACATGAGCGTAGCCAG	0.043 ± 0.002
gTTgACATGAGCGTAGCCAG	0.045 ± 0.004
AgTAACAaGAGCGTAGCCAG	0.045 ± 0.002
AaTAACcTGAGCGTAGCCAG	0.050 ± 0.002
AcTAACAaGAGCGTAGCCAG	0.050 ± 0.002
AcTAACAgGAGCGTAGCCAG	0.051 ± 0.002
AgTAACAgGAGCGTAGCCAG	0.051 ± 0.002
tTTAtCATGAGCGTAGCCAG	0.052 ± 0.002
tTTtACATGAGCGTAGCCAG	0.053 ± 0.026
AaTtACATGAGCGTAGCCAG	0.053 ± 0.002
cTTAACgTGAGCGTAGCCAG	0.053 ± 0.016
AcTAACgTGAGCGTAGCCAG	0.054 ± 0.003
cTTAACAaGAGCGTAGCCAG	0.054 ± 0.002
cTTAAgATGAGCGTAGCCAG	0.055 ± 0.015
AaTAAgATGAGCGTAGCCAG	0.056 ± 0.002
AcTAACATtAGCGTAGCCAG	0.057 ± 0.003
tTTAAaATGAGCGTAGCCAG	0.057 ± 0.001
cTTAACAgGAGCGTAGCCAG	0.058 ± 0.008
cTTAACATcAGCGTAGCCAG	0.059 ± 0.001

cTTAACATtAGCGTAGCCAG	0.060 ± 0.002
AgTAACgTGAGCGTAGCCAG	0.061 ± 0.003
gTTAACgTGAGCGTAGCCAG	0.061 ± 0.004
tTTAACgTGAGCGTAGCCAG	0.061 ± 0.005
AgTAACATtAGCGTAGCCAG	0.062 ± 0.002
AcTAACATGAGCGTAGCgAG	0.063 ± 0.002
AcTAACATcAGCGTAGCCAG	0.064 ± 0.003
AgTAACAcGAGCGTAGCCAG	0.064 ± 0.002
AgTAACATcAGCGTAGCCAG	0.066 ± 0.003
cTTAACATGAGaGTAGCCAG	0.067 ± 0.002
cTTAACAcGAGCGTAGCCAG	0.068 ± 0.002
tTTAACAgGAGCGTAGCCAG	0.068 ± 0.002
cTTAACATGAGCGTAGCgAG	0.069 ± 0.002
ATaAcCATGAGCGTAGCCAG	0.070 ± 0.004
tTTAACAaGAGCGTAGCCAG	0.070 ± 0.002
tTTAAgATGAGCGTAGCCAG	0.071 ± 0.002
cTTAACATGtGCGTAGCCAG	0.071 ± 0.001
AaTAAaATGAGCGTAGCCAG	0.071 ± 0.015
ATgAcCATGAGCGTAGCCAG	0.073 ± 0.004
tTTAACATtAGCGTAGCCAG	0.073 ± 0.002
cTTAACATaAGCGTAGCCAG	0.073 ± 0.002
cTTAACATGAGCGcAGCCAG	0.073 ± 0.002
AcTAACAcGAGCGTAGCCAG	0.073 ± 0.003
AgTAACATaAGCGTAGCCAG	0.074 ± 0.003
AgTAACATGAGCGTAGCgAG	0.074 ± 0.003
cTTAACATGAGCGTAGCtAG	0.076 ± 0.002
AcTAACATGgGCGTAGCCAG	0.076 ± 0.004
AaTAACtTGAGCGTAGCCAG	0.076 ± 0.003
cTTAACATGAGCGTAGCCAt	0.078 ± 0.002
cTTAACATGAGCGTgGCCAG	0.078 ± 0.004
cTTAACATGAGCGTAGCCtG	0.078 ± 0.002
AgTAACATGgGCGTAGCCAG	0.078 ± 0.003
AcTAACATaAGCGTAGCCAG	0.078 ± 0.004
AcTAACATGtGCGTAGCCAG	0.080 ± 0.003
gTTAACAgGAGCGTAGCCAG	0.081 ± 0.005
cTTAACATGAGtGTAGCCAG	0.081 ± 0.002
cTTAACATGAtCGTAGCCAG	0.081 ± 0.002
AgTAACATGAGCGcAGCCAG	0.082 ± 0.003
gTTAACATtAGCGTAGCCAG	0.083 ± 0.006
AcTAACATGAGCGcAGCCAG	0.083 ± 0.003
AcTAACATGAGCGTAGCtAG	0.083 ± 0.003
cTTAACATGAGCaTAGCCAG	0.084 ± 0.003
cTTAACATGAGCGTcGCCAG	0.084 ± 0.002
cTTAACATGcGCGTAGCCAG	0.084 ± 0.003
cTTAACATGAGCGTAGCCAG	0.085 ± 0.002

AgTAACATGtGCGTAGCCAG	0.085 ± 0.003
AcTAACATGAGCGgAGCCAG	0.086 ± 0.003
tTTAACATcAGCGTAGCCAG	0.086 ± 0.003
cTTAACATGgGCGTAGCCAG	0.086 ± 0.011
cTTAACATGAGCGgAGCCAG	0.087 ± 0.002
AcTAACATGAGaGTAGCCAG	0.087 ± 0.003
cTTAACATGAGgGTAGCCAG	0.087 ± 0.002
gTTAACATGAGCGTAGCgAG	0.087 ± 0.004
AcTAACATGAGCGTAGCCAG	0.087 ± 0.003
AgTAACATGAGaGTAGCCAG	0.088 ± 0.003
cTTAACATGAGCGTAGCCgG	0.088 ± 0.003
tTTAACATGgGCGTAGCCAG	0.088 ± 0.002
cTTAACATGAGCGTAGCaAG	0.088 ± 0.002
AcTAACATGAGCGTgGCCAG	0.088 ± 0.004
AcTAACATGAGCGTAGCCAt	0.088 ± 0.004
cTTAACATGAGCGaAGCCAG	0.088 ± 0.003
cTTAACATGAGCGTAGgCAG	0.088 ± 0.003
cTTAACATGAGCGTAGCCcG	0.089 ± 0.002
cTTAACATGAGCGTtGCCAG	0.089 ± 0.003
AcTAACATGAGCGTAGtCAG	0.089 ± 0.004
cTTAACATGAGCGTAGtCAG	0.090 ± 0.005
AcTAACATGAGCGTcGCCAG	0.090 ± 0.004
cTTAACATGAGCtTAGCCAG	0.091 ± 0.003
AgTAACATGAGCGTAGCtAG	0.091 ± 0.003
cTTAACATGAGCGTAGCCAa	0.091 ± 0.002
AcTAACATGAGCGTtGCCAG	0.091 ± 0.004
AcTAACATGAGCaTAGCCAG	0.092 ± 0.004
tTTAACATGAGCGTAGCgAG	0.092 ± 0.004
tTTAACATaAGCGTAGCCAG	0.092 ± 0.002
cTTAACATGAGCGTAGaCAG	0.092 ± 0.004
cTTAACATGAGCcTAGCCAG	0.092 ± 0.002
AcTAACATGAGCtTAGCCAG	0.092 ± 0.003
AgTAACATGAGCGaAGCCAG	0.093 ± 0.003
AcTAACATGAGCGTAGCCtG	0.093 ± 0.004
AcTAACATGcGCGTAGCCAG	0.093 ± 0.005
cTTAACATGAGCGTAtCCAG	0.093 ± 0.002
AcTAACATGAGCGTAtCCAG	0.094 ± 0.004
tTTAACAcGAGCGTAGCCAG	0.094 ± 0.003
AgTAACATGAGCaTAGCCAG	0.094 ± 0.004

c

**Sequence**	**Normalized fluorescence intensity±SEM**
AcCATATCCGTGTCGCCCTT	1.022 ± 0.012

AACATATCCGTGTCGCCCTT	1.000 ± 0.035
AtCATATCCGTGTCGCCCTT	0.998 ± 0.010
tACATATCCGTGTCGCCCTT	0.996 ± 0.017
cACATATCCGTGTCGCCCTT	0.995 ± 0.026
gACATATCCGTGTCGCCCTT	0.986 ± 0.015
gcCATATCCGTGTCGCCCTT	0.973 ± 0.006
tgCATATCCGTGTCGCCCTT	0.971 ± 0.011
AcaATATCCGTGTCGCCCTT	0.971 ± 0.009
AcCATATCCGTGTtGCCCTT	0.970 ± 0.014
gACATATCCGTGTtGCCCTT	0.970 ± 0.013
AAtATATCCGTGTCGCCCTT	0.970 ± 0.010
AtCATATCCGTGTCcCCCTT	0.966 ± 0.009
tACATATCCGTGTCcCCCTT	0.966 ± 0.012
AtaATATCCGTGTCGCCCTT	0.963 ± 0.008
AACATATCCGTGcCGCCCTT	0.963 ± 0.013
gtCATATCCGTGTCGCCCTT	0.962 ± 0.016
ccCATATCCGTGTCGCCCTT	0.961 ± 0.020
tAtATATCCGTGTCGCCCTT	0.960 ± 0.012
ctCATATCCGTGTCGCCCTT	0.959 ± 0.028
cAtATATCCGTGTCGCCCTT	0.958 ± 0.014
AACATATCCGTGTCcCCCTT	0.957 ± 0.012
AcCtTATCCGTGTCGCCCTT	0.957 ± 0.011
AgCATATCCGTGTCGCCCTT	0.956 ± 0.027
cACtTATCCGTGTCGCCCTT	0.952 ± 0.013
AgaATATCCGTGTCGCCCTT	0.951 ± 0.013
AACATATCCGTGTCaCCCTT	0.950 ± 0.012
AcCATATCCGTGTCcCCCTT	0.948 ± 0.010
tACtTATCCGTGTCGCCCTT	0.948 ± 0.010
tACATATCCGTtTCGCCCTT	0.945 ± 0.010
tACATATCCGTGTtGCCCTT	0.944 ± 0.018
cACATATCCGTGTtGCCCTT	0.943 ± 0.031
tcCATATCCGTGTCGCCCTT	0.943 ± 0.015
AgCATATCCGTGTtGCCCTT	0.942 ± 0.014
tACATATCCGTGcCGCCCTT	0.940 ± 0.009
AACAaATCCGTGTCcCCCTT	0.939 ± 0.025
AACtTATCCGTGTCGCCCTT	0.939 ± 0.031
cACATATCCGTGcCGCCCTT	0.938 ± 0.012
cACcTATCCGTGTCGCCCTT	0.937 ± 0.008
cAaATATCCGTGTCGCCCTT	0.936 ± 0.012
AtCATATCCGTGTtGCCCTT	0.935 ± 0.034
ttCATATCCGTGTCGCCCTT	0.935 ± 0.019
AACATATCCGTGaCGCCCTT	0.934 ± 0.012
cACATATCCGTGTCcCCCTT	0.934 ± 0.014
AAtcTATCCGTGTCGCCCTT	0.932 ± 0.010
cACATATCCGTGTCaCCCTT	0.932 ± 0.009

tACATATCCGTGTCtCCCTT	0.932 ± 0.011
AgCATATCCGTGTCcCCCTT	0.930 ± 0.015
cACATATCCGTtTCGCCCTT	0.929 ± 0.011
AAatTATCCGTGTCGCCCTT	0.929 ± 0.013
AACATATCCGTtTCGCCCTT	0.928 ± 0.028
AACtTATCCGTGTtGCCCTT	0.928 ± 0.009
AcCcTATCCGTGTCGCCCTT	0.927 ± 0.007
AACATATCCGTGTCtCCCTT	0.926 ± 0.009
AttATATCCGTGTCGCCCTT	0.926 ± 0.023
gACATATCCGTGcCGCCCTT	0.925 ± 0.012
cACATATCCGTGTaGCCCTT	0.925 ± 0.010
AACATATCCGTGTCGtCCTT	0.923 ± 0.012
tAaATATCCGTGTCGCCCTT	0.923 ± 0.022
tACATATCCGTGTCaCCCTT	0.921 ± 0.011
gACATATCCGTGTCcCCCTT	0.921 ± 0.011
AAgcTATCCGTGTCGCCCTT	0.920 ± 0.012
AACATATCCGTGTtGCCCTT	0.919 ± 0.045
AACATATCCGTaTCGCCCTT	0.919 ± 0.011
AACATccCCGTGTCGCCCTT	0.919 ± 0.010
AACATATCCGTcTCGCCCTT	0.918 ± 0.009
ggCATATCCGTGTCGCCCTT	0.918 ± 0.028
AACATATCCGTGTgcCCCTT	0.918 ± 0.011
AACATATCCGTGTaGCCCTT	0.917 ± 0.010
AcCATATCCGTtTCGCCCTT	0.916 ± 0.012
tACATATCCGTGTaGCCCTT	0.915 ± 0.013
AACcTATCCGTGTCGCCCTT	0.914 ± 0.014
gACATATCCGTtTCGCCCTT	0.914 ± 0.014
AgCcTATCCGTGTCGCCCTT	0.912 ± 0.011
AAaATAcCCGTGTCGCCCTT	0.912 ± 0.010
AACcaATCCGTGTCGCCCTT	0.911 ± 0.013
AtCAaATCCGTGTCGCCCTT	0.911 ± 0.011
AtCATATCCGTtTCGCCCTT	0.911 ± 0.013
AAtATATCCGTGTCcCCCTT	0.911 ± 0.013
AACtaATCCGTGTCGCCCTT	0.910 ± 0.017
AAtAaATCCGTGTCGCCCTT	0.910 ± 0.024
AAaATATCCGTGTCcCCCTT	0.910 ± 0.024
tACATATCCGTcTCGCCCTT	0.908 ± 0.009
AtCATATCCGTGTCtCCCTT	0.908 ± 0.010
tACAaATCCGTGTCGCCCTT	0.907 ± 0.011
AACATATCCGTGTtcCCCTT	0.907 ± 0.009
cACATATCCGTaTCGCCCTT	0.907 ± 0.011
AtCATATCCGTGTaGCCCTT	0.907 ± 0.012
AAgATATCCGTGTCGCCCTT	0.906 ± 0.011
AAaATATCCGTGTtGCCCTT	0.906 ± 0.011
AACtTATCCGTGTaGCCCTT	0.905 ± 0.016

AAaAaATCCGTGTCGCCCTT	0.905 ± 0.010
AcCATATCCGTGcCGCCCTT	0.903 ± 0.013
AAtATATCCGTGTaGCCCTT	0.903 ± 0.010
AgCtTATCCGTGTCGCCCTT	0.903 ± 0.026
AtCATATCCGTGcCGCCCTT	0.902 ± 0.028
tACcTATCCGTGTCGCCCTT	0.902 ± 0.013
AtCAcATCCGTGTCGCCCTT	0.902 ± 0.010
cACATATCCcTGTCGCCCTT	0.902 ± 0.014
cAgATATCCGTGTCGCCCTT	0.901 ± 0.015
AAtATcTCCGTGTCGCCCTT	0.901 ± 0.013
AACATATCCGTccCGCCCTT	0.901 ± 0.010
AAaAcATCCGTGTCGCCCTT	0.901 ± 0.013
gAaATATCCGTGTCGCCCTT	0.900 ± 0.043
gACATATCCGTaTCGCCCTT	0.900 ± 0.011
AtgATATCCGTGTCGCCCTT	0.900 ± 0.014
AACcTgTCCGTGTCGCCCTT	0.899 ± 0.012
AAaATATCCGTGTCGCCCTT	0.899 ± 0.036
tAgATATCCGTGTCGCCCTT	0.899 ± 0.012
gACATATCCGTGTCaCCCTT	0.899 ± 0.008
AACATAcCCGTGTCcCCCTT	0.899 ± 0.014
AAtgTATCCGTGTCGCCCTT	0.899 ± 0.019
AACATATCCtTGTCGCCCTT	0.898 ± 0.011
AtCATATCCGTcTCGCCCTT	0.898 ± 0.009
cACATATCCGTGaCGCCCTT	0.897 ± 0.014
AAgtTATCCGTGTCGCCCTT	0.897 ± 0.012
AACATtcCCGTGTCGCCCTT	0.897 ± 0.012
AAaATAgCCGTGTCGCCCTT	0.896 ± 0.016
AACcTAcCCGTGTCGCCCTT	0.896 ± 0.011
cgCATATCCGTGTCGCCCTT	0.896 ± 0.045
AAttTATCCGTGTCGCCCTT	0.896 ± 0.023
AACATATCCGctTCGCCCTT	0.895 ± 0.010
AACATgTCCGTGTCcCCCTT	0.895 ± 0.013
AcCAcATCCGTGTCGCCCTT	0.895 ± 0.011
tACATtTCCGTGTCGCCCTT	0.895 ± 0.012
tACATATCCGTaTCGCCCTT	0.895 ± 0.013
AACATATCtGTGTCGCCCTT	0.895 ± 0.011
AAtAcATCCGTGTCGCCCTT	0.894 ± 0.022
AAaATAaCCGTGTCGCCCTT	0.894 ± 0.009
AcCATATCCGTaTCGCCCTT	0.894 ± 0.009
AAgATATCCGTGTCcCCCTT	0.893 ± 0.010
cACAaATCCGTGTCGCCCTT	0.893 ± 0.011
AgCATATCCGTGcCGCCCTT	0.893 ± 0.011
cACATATCCGTGTCtCCCTT	0.893 ± 0.022
AAaATcTCCGTGTCGCCCTT	0.893 ± 0.014
AtCATATCCGTaTCGCCCTT	0.892 ± 0.010

AAtATAaCCGTGTCGCCCTT	0.892 ± 0.014
AACATAcCCGTGTCGCCCTT	0.892 ± 0.009
AACATtTCCGTGTCcCCCTT	0.892 ± 0.025
AACATcTCaGTGTCGCCCTT	0.891 ± 0.016
AgCATATCCGTGTCtCCCTT	0.891 ± 0.011
AcCATATCCGTGTaGCCCTT	0.891 ± 0.041
AACATcTCCtTGTCGCCCTT	0.891 ± 0.030
gACATATCCGTGTaGCCCTT	0.890 ± 0.014
AtCATATCCGTGTCaCCCTT	0.890 ± 0.023
AACccATCCGTGTCGCCCTT	0.890 ± 0.010
AACgTATCCGTGTCcCCCTT	0.889 ± 0.009
AACATATCCGTtcCGCCCTT	0.889 ± 0.028
AtCATtTCCGTGTCGCCCTT	0.889 ± 0.009
AACATcTCCGTGTCGCCCTT	0.888 ± 0.008
AACAcATCCGTGTCGCCCTT	0.888 ± 0.013
AACtTtTCCGTGTCGCCCTT	0.888 ± 0.011
cACATtTCCGTGTCGCCCTT	0.888 ± 0.011
gACcTATCCGTGTCGCCCTT	0.888 ± 0.016
AcCATATCCGTcTCGCCCTT	0.887 ± 0.010
AcCATcTCCGTGTCGCCCTT	0.887 ± 0.010
AAgATATCCGTGTaGCCCTT	0.886 ± 0.012
AAgATAcCCGTGTCGCCCTT	0.886 ± 0.010
AgtATATCCGTGTCGCCCTT	0.886 ± 0.014
AACATATCCGTtaCGCCCTT	0.886 ± 0.011
AACATATCCGgtTCGCCCTT	0.886 ± 0.011
AgCATgTCCGTGTCGCCCTT	0.886 ± 0.011
AACcTATCCcTGTCGCCCTT	0.886 ± 0.011
AcCATATCCGTGaCGCCCTT	0.885 ± 0.011
AcCATtTCCGTGTCGCCCTT	0.885 ± 0.012
AACcTATCCGTGTCcCCCTT	0.885 ± 0.027
AACATATCCtgGTCGCCCTT	0.885 ± 0.011
AACAaATCCGTGTaGCCCTT	0.885 ± 0.013
AACAaAcCCGTGTCGCCCTT	0.885 ± 0.011
AACATgTCCGTGTCGCCCTT	0.885 ± 0.008
cACATATCCGTcTCGCCCTT	0.885 ± 0.015
AACtTcTCCGTGTCGCCCTT	0.885 ± 0.013
AACATcTgCGTGTCGCCCTT	0.885 ± 0.017
AACATATCCGgGTCGCCCTT	0.885 ± 0.011
AtCATgTCCGTGTCGCCCTT	0.885 ± 0.010
AAtATATCCGTGcCGCCCTT	0.884 ± 0.012
AtCcTATCCGTGTCGCCCTT	0.884 ± 0.020

d

Sequence	Normalized fluorescence intensity±SEM
AACATATgCGTGTCGCCCTg	0.022 ± 0.001
AACAaATCCGTGTCGCCCTg	0.022 ± 0.001
AACATtTCCGTGTCGCCCTg	0.024 ± 0.001
AACATATCCaTGTCGCCCTg	0.024 ± 0.001
AACATATCCGgGTCGCCCTg	0.025 ± 0.001
AACATATCCGTGgCGCCCTg	0.025 ± 0.001
AACATATCgGTGTCGCCCTg	0.025 ± 0.001
AACATAgCCGTGTCGCCCTg	0.026 ± 0.001
AAaATATCCGTGTCGCCCTg	0.026 ± 0.001
AACATATCaGTGTCGCCCTg	0.026 ± 0.001
AACATATCCGTGTgGCCCTg	0.027 ± 0.001
AACATATCCcTGTCGCCCTg	0.027 ± 0.001
AACATATCCGTtTCGCCCTg	0.029 ± 0.001
AACATATCCGTGTtGCCCTg	0.031 ± 0.001
AACATgTCCGTGTCGCCCTg	0.031 ± 0.001
AACATATCCGTGTCGCCCaa	0.031 ± 0.001
AACATATCCGTGcCGCCCTg	0.032 ± 0.001
AACATAaCCGTGTCGCCCTg	0.032 ± 0.001
AACATAcCCGTGTCGCCCTg	0.032 ± 0.002
AACATcTCCGTGTCGCCCTg	0.033 ± 0.001
AACAgATCCGTGTCGCCCTg	0.033 ± 0.001
AACATATaCGTGTCGCCCTg	0.034 ± 0.001
AACAaATCCGTGTCGCCCTa	0.035 ± 0.001
AACAcATCCGTGTCGCCCTg	0.035 ± 0.001
AACATATCCGTGTCGCCCgg	0.035 ± 0.001
AACATATCCtTGTCGCCCTg	0.036 ± 0.002
AACATATgCGTGTCGCCCTa	0.036 ± 0.002
AACATATCCGaGTCGCCCTg	0.037 ± 0.001
AACATtTCCGTGTCGCCCTa	0.037 ± 0.001
AAgATATCCGTGTCGCCCTg	0.038 ± 0.002
AACATATCCGcGTCGCCCTg	0.038 ± 0.001
AACcTATCCGTGTCGCCCTg	0.039 ± 0.002
AACATATtCGTGTCGCCCTg	0.040 ± 0.001
AACATATCtGTGTCGCCCTg	0.040 ± 0.001
AAaATATCCGTGTCGCCCTa	0.040 ± 0.001
AACATATCCaTGTCGCCCTa	0.040 ± 0.002
AACATATCCcTGTCGCCCTa	0.041 ± 0.002
AACATATCCGgGTCGCCCTa	0.041 ± 0.003
AACATATCCGTGaCGCCCTg	0.043 ± 0.002
AACATATCCGTGgCGCCCTa	0.043 ± 0.003
AACATATCCGTcTCGCCCTg	0.044 ± 0.002
AACATATCCGTGTCGCCCag	0.044 ± 0.002
AACATATCCGTaTCGCCCTg	0.044 ± 0.001
AACATAgCCGTGTCGCCCTa	0.045 ± 0.001

AAtATATCCGTGTCGCCCTg	0.045 ± 0.001
AACtTATCCGTGTCGCCCTg	0.045 ± 0.001
AtCATATCCGTGTCGCCCTg	0.047 ± 0.003
AACATATCCGTGTgGCCCTa	0.047 ± 0.002
AACATATCCGTGTaGCCCTg	0.048 ± 0.002
AACgTATCCGTGTCGCCCTg	0.048 ± 0.001
AgCATATCCGTGTCGCCCTg	0.049 ± 0.002
cACATATCCGTGTCGCCCTg	0.050 ± 0.003
AACATATCCGTGTCaCCCTg	0.050 ± 0.002
AACATATCaGTGTCGCCCTa	0.050 ± 0.001
AACATATCgGTGTCGCCCTa	0.050 ± 0.001
gACATATCCGTGTCGCCCTg	0.050 ± 0.002
AACATATCCtTGTCGCCCTa	0.050 ± 0.002
AACATATCCGTGTCGCCCTg	0.051 ± 0.003
AcCATATCCGTGTCGCCCTg	0.051 ± 0.001
tACATATCCGTGTCGCCCTg	0.052 ± 0.003
AACATATCCGTtTCGCCCTa	0.052 ± 0.002
AACATATCCGTGTCtCCCTg	0.053 ± 0.001
AACATATCCGTGTCGCCCga	0.053 ± 0.003
AACATATCCGTGcCGCCCTa	0.053 ± 0.002
AACATATCCGTGTtGCCCTa	0.053 ± 0.003
AACATAaCCGTGTCGCCCTa	0.054 ± 0.002
AACATcTCCGTGTCGCCCTa	0.056 ± 0.002
AACATATCCGTGTCGCgCTg	0.056 ± 0.003
AACATATCCGTGTCGgCCTg	0.057 ± 0.002
AACATgTCCGTGTCGCCCTa	0.057 ± 0.002
AACATATaCGTGTCGCCCTa	0.060 ± 0.001
AACATAcCCGTGTCGCCCTa	0.061 ± 0.002
AACATATCCGTGTCGaCCTg	0.061 ± 0.002
AACAgATCCGTGTCGCCCTa	0.061 ± 0.002
AACAcATCCGTGTCGCCCTa	0.063 ± 0.003
AACATATCCGTGTCGCCaTg	0.065 ± 0.003
AACATATCCGTGTCGCtCTg	0.066 ± 0.001
AACATATCCGTGTCGCaCTg	0.066 ± 0.003
AACATATCCGTGTCcCCCTg	0.068 ± 0.003
AACATATCtGTGTCGCCCTa	0.069 ± 0.003
AACATATCCGaGTCGCCCTa	0.069 ± 0.002
AAgATATCCGTGTCGCCCTa	0.069 ± 0.002
AACATATCCGcGTCGCCCTa	0.071 ± 0.002
AACATATCCGTGTCGCCgTg	0.073 ± 0.002
AACATATtCGTGTCGCCCTa	0.073 ± 0.003
AACATATCCGTGTCGCCCgc	0.073 ± 0.002
AACcTATCCGTGTCGCCCTa	0.076 ± 0.002
AACtTATCCGTGTCGCCCTa	0.078 ± 0.004
AACATATCCGTGTCGCCCca	0.078 ± 0.004

AACATATCCGTGTCGtCCTg	0.079 ± 0.002
AACATATCCGTGaCGCCCTa	0.083 ± 0.005
AAtATATCCGTGTCGCCCTa	0.083 ± 0.004
AACATATCCGTaTCGCCCTa	0.083 ± 0.004
AACATATCCGTcTCGCCCTa	0.084 ± 0.003
AACATATCCGTGTCGCCCcc	0.084 ± 0.005
AACATATCCGTGTCGCCgTa	0.086 ± 0.003
AACgTATCCGTGTCGCCCTa	0.087 ± 0.002
AACATATgCGTGTCGCCCTc	0.087 ± 0.005
AACATATCCGTGTaGCCCTa	0.088 ± 0.005
AACATATCCGTGTCtCCCTa	0.089 ± 0.003
AACATATCCGTGTCcCCCTa	0.090 ± 0.002
AgCATATCCGTGTCGCCCTa	0.091 ± 0.003
AACATATCCGTGTCGCCCac	0.092 ± 0.004
AACATATCCGTGTCaCCCTa	0.094 ± 0.003
gACATATCCGTGTCGCCCTa	0.096 ± 0.004
tACATATCCGTGTCGCCCTa	0.097 ± 0.006
AACATATCCGTGTCGCCCcg	0.098 ± 0.004
AACATATCCGTGTCGCCCTa	0.099 ± 0.004
cACATATCCGTGTCGCCCTa	0.100 ± 0.003
AcCATATCCGTGTCGCCCTa	0.100 ± 0.002
AtCATATCCGTGTCGCCCTa	0.103 ± 0.003
AACATATCCGTGTCGCCaTa	0.104 ± 0.003
AACATATCCGTGTCGgCCTa	0.105 ± 0.004
AACATATCCGTGTCGCgCTa	0.108 ± 0.002
AACATATCCGTGTCGaCCTa	0.114 ± 0.002
AACATATCCGTGTCGCaCTc	0.116 ± 0.006
AACATATCCGTGTCGCCgTc	0.116 ± 0.006
AACATATCCGTGTCGCtCTa	0.117 ± 0.002
AACATAgCCGTGTCGCCCTc	0.118 ± 0.003
AACATATaCGTGTCGCCCTc	0.126 ± 0.007
AACATATCCGTGTCGCCtTg	0.126 ± 0.002
AACATATCaGTGTCGCCCTc	0.127 ± 0.006
AACATATCCGTGTCGCCaTc	0.128 ± 0.007
AACATATCCGTGTCGCaCTa	0.128 ± 0.003
AACATATCCGTGTCGtCCTa	0.130 ± 0.004
AACATATCgGTGTCGCCCTc	0.132 ± 0.006
AACATgTCCGTGTCGCCCTc	0.141 ± 0.012
AACATAaCCGTGTCGCCCTc	0.149 ± 0.005
AACATATCCGTGTCGCCtTa	0.154 ± 0.006
AACATATCCGTGTCGCgCTc	0.154 ± 0.005
AACAaATCCGTGTCGCCCTc	0.159 ± 0.005
AACATcTCCGTGTCGCCCTc	0.162 ± 0.004
AACAgATCCGTGTCGCCCTc	0.164 ± 0.008
AACATATCCGTGTCGCtCTc	0.170 ± 0.002

AACATAcCCGTGTCGCCCTc	0.170 ± 0.002
AACATATCCGaGTCGCCCTc	0.178 ± 0.010
AACATtTCCGTGTCGCCCTc	0.183 ± 0.005
AACAcATCCGTGTCGCCCTc	0.183 ± 0.005
AACATATCCGTGTCGCCtTc	0.186 ± 0.003
AAaATATCCGTGTCGCCCTc	0.192 ± 0.008
AACATATCCGgGTCGCCCTc	0.193 ± 0.013
AACATATCCGcGTCGCCCTc	0.194 ± 0.003
AACATATCCaTGTCGCCCTc	0.195 ± 0.003
AACATATCCGTGTCGgCCTc	0.199 ± 0.012
AACATATCCcTGTCGCCCTc	0.201 ± 0.007
AACATATCCtTGTCGCCCTc	0.204 ± 0.004
AACgTATCCGTGTCGCCCTc	0.204 ± 0.011
AACcTATCCGTGTCGCCCTc	0.207 ± 0.004
AACATATCCGTcTCGCCCTc	0.212 ± 0.009
AACATATCCGTGTCGaCgTT	0.216 ± 0.004
AACATATtCGTGTCGCCCTc	0.219 ± 0.011
AACATATCtGTGTCGCCCTc	0.219 ± 0.008
AAgATATCCGTGTCGCCCTc	0.224 ± 0.006
AACATATCCGTGgCGCCCTc	0.226 ± 0.011
AACATATCCGTGTgGCCCTc	0.227 ± 0.011
AACATATCCGTGTCGaCCTc	0.231 ± 0.004
AACATATCCGTGTCtCCCTc	0.234 ± 0.009
AACATATCCGTtTCGCCCTc	0.240 ± 0.012
AACATATCCGTGTCGCggTT	0.244 ± 0.013
AACATATCCGTaTCGCCCTc	0.248 ± 0.009
AACATATCCGTGaCGCCCTc	0.257 ± 0.012
AACATATCCGTGTCGaCaTT	0.258 ± 0.004
AACtTATCCGTGTCGCCCTc	0.261 ± 0.006
AACATATCCGTGTCGCgCgT	0.261 ± 0.005
AACATATCCGTGTCGgCgTT	0.264 ± 0.005
AACATATgCGTGTCGCCCgT	0.265 ± 0.009
AACATATCCGTGcCGCCCTc	0.267 ± 0.008
AAtATATCCGTGTCGCCCTc	0.274 ± 0.008
AACATATCCGTGTCcCCCTc	0.274 ± 0.009
AACATATCCGTGTtGCCCTc	0.277 ± 0.006
AACATATgCGTGTCGCCgTT	0.284 ± 0.009
AACATATCCGTGTaGCCCTc	0.285 ± 0.007
AACATATCCGTGTCGCaCgT	0.294 ± 0.007
AACATATCCGTGTCGCgaTT	0.295 ± 0.003
AACATATCCGTGTCaCCCTc	0.296 ± 0.009
AACATATgCGTGTCGCaCTT	0.301 ± 0.005
AtCATATCCGTGTCGCCCTc	0.303 ± 0.013

Supplementary Table S4. Logarithmic brightness image decomposition was used to generate virtual masks for high-complexity post-synthetic surface modification. The greyscale image′s bit depth was reduced from 8 bits (256 shades of grey) to 38 shades of grey. To account for higher sensitivity to changes in the darker areas of the image, the level spacing was chosen to be approximately logarithmic, with closer spacing between darker grey.

**Mask**	**Original intensity range**	**Selected range**	**Delta**	**Intensity value**	**Cumulative exposure time (s)**	**Exposure time for this mask for 9 mW/cm²**
none	0-255		20	255	0	
1	0-235	235-255	20	235	11	11
2	0-215	215-255	20	215	23	12
3	0-195	195-255	20	195	37	14
4	0-175	175-255	20	175	52	15
5	0-155	155-255	20	155	68	16
6	0-135	135-255	20	135	87	19
7	0-115	115-255	20	115	110	23
8	0-95	95-255	20	95	136	26
9	0-75	75-255	20	75	169	30
10	0-60	60-255	15	60	200	31
11	0-45	45-255	15	45	240	40
12	0-35	35-255	10	35	275	35
13	0-30	30-255	5	30	296	21
14	0-26	26-255	4	26	316	20
15	0-24	24-255	2	24	326	10
16	0-22	23-255	1	23	333	7
17	0-21	22-255	1	22	338	5
18	0-20	21-255	1	21	345	7
19	0-19	20-	1	20	352	7
20	0-18	19	1	19	359	7
21	0-17	18	1	18	367	8
22	0-16	17	1	17	375	8
23	0-15	16	1	16	383	8
24	0-14	15	1	15	392	9
25	0-13	14	1	14	402	10
26	0-12	13	1	13	412	10
27	0-11	12	1	12	423	11
28	0-10	11	1	11	434	11
29	0-9	10	1	10	448	14
30	0-8	9	1	9	462	14
31	0-7	8	1	8	479	17
32	0-6	7	1	7	497	18
33	0-5	6	1	6	519	22
34	0-4	5	1	5	544	25
35	0-3	4	1	4	576	32
36	0-2	3	1	3	614	38
37	0-1	2	1	2	671	57
38	0-1	1	1	1	746	75
